# Role of Earth system processes in the relationship between climate change and cumulative carbon emissions

**DOI:** 10.1038/s41467-026-72930-7

**Published:** 2026-05-19

**Authors:** Spencer K. Liddicoat, Timothy Andrews, Chris D. Jones, Lina M. Mercado, Mark A. Ringer, Eddy Robertson, Stephen Sitch, Andy Wiltshire

**Affiliations:** 1 Met Office, FitzRoy Road, Exeter, Devon, UK; 2 Faculty of Environment, Science and Economy, University of Exeter, North Park Road, Exeter, UK; 3 School of Earth and Environment, University of Leeds, Leeds, UK; 4 School of Geographical Sciences, University of Bristol, Bristol, UK; 5 UK CEH Centre for Ecology and Hydrology, Wallingford, UK

**Keywords:** Climate and Earth system modelling, Carbon cycle

## Abstract

Estimates of carbon emissions budgets to limit global warming to 1.5 °C or 2 °C rely on the near-linear relationship between global temperature change and total CO_2_ emitted, known as the Transient Climate Response to cumulative CO_2_ Emissions (TCRE). The TCRE is determined from Earth System Models (ESMs) and is therefore sensitive to the physical and biogeochemical processes represented within them. Here we use an ESM (UKESM) to explore the sensitivity of TCRE to six Earth system processes in isolation. Four processes increase TCRE: fire-vegetation interactions by 14.6%; nitrogen limitation of vegetation by 9.7%; diffuse radiation effects on vegetation by 8.5%; and interactive emissions of methane from wetlands by 5.1%. Conversely, two processes marginally reduce TCRE: allowing the vegetation distribution to adapt to changing climate and CO_2_ lowers TCRE by 1.5%, and climate impacts from the emission of biogenic volatile organic compounds reduce it by 1.4%. We demonstrate the extent to which each process changes TCRE via its influence on the climate and on the global carbon cycle, and discuss underlying mechanisms. Our results highlight the substantial process-dependence of model-derived estimates of TCRE, with implications for remaining carbon budgets to future warming targets calculated from them.

## Abstract (web summary)

Models reveal that 4 Earth system processes, including fire and nutrient limitation of vegetation, increase the amount of global warming per unit of CO2 emitted by ≤15%, while vegetation redistribution and BVOC emissions reduce it by around 1.5%.

## Introduction

The transient climate response to cumulative CO_2_ emissions (TCRE) is an important metric of the near-linear relationship between future planetary warming and cumulative emissions (CE) of carbon dioxide^1,2^. It provides policymakers with a framework for estimating the level at which total cumulative CO_2_ emissions must be capped in future to limit warming to the Paris Agreement targets of 1.5 °C or 2 °C above pre-industrial. Since historical CO_2_ emissions are known^3^, the remaining carbon budget (RCB) for a future warming target can be inferred. The TCRE is determined using global Earth system models (ESMs), which calculate the flows of energy, moisture and carbon between the ocean, the atmosphere, and the terrestrial biosphere. ESMs simulate the temperature response to a prescribed CO_2_ concentration pathway, and the CO_2_ emissions compatible with this pathway can be diagnosed; the TCRE is derived from these two quantities, typically when atmospheric CO_2_ concentration reaches double the pre-industrial (1850) level. Many ESMs of varying complexity participate in the Coupled Model Intercomparison Project (CMIP), a global endeavour to further our understanding of the climate and carbon cycle. The ESMs of CMIP's sixth phase (CMIP6) comprise an ‘ensemble of opportunity’ for policymakers, providing a distribution of TCRE across the mix of processes in the ESMs; however, this is inevitably a subset of the full range of Earth system processes known to influence the climate and carbon cycle, limiting the possibility of a complete understanding of TCRE and its uncertainty. To date, no systematic study into the process-dependence of TCRE, as envisioned by Matthews et al. (2020) ^4^, has appeared in the literature since its inception in 2009.

Here, we present a framework to interpret the TCRE in a mixed process ensemble, using results from the UK Earth System Model (UKESM) to assess the influence on TCRE of six Earth system processes. Firstly, nitrogen limitation of land carbon uptake: nitrogen is a limiting nutrient which can reduce the fertilising effect of rising CO_2_ on plant productivity and growth^5^, so excluding this process can lead to an overestimate of the land carbon sink. Second, fire-vegetation interactions: fire reduces aboveground biomass^6,7^, reducing carbon storage^8^, and lowering photosynthetic potential^9^. Third, dynamic vegetation: simulating competition between trees, shrubs and grasses allows their distribution to evolve in response to changing climate and CO_2_, thereby influencing land carbon uptake. Fourth, diffuse radiation impacts on vegetation: a greater diffuse radiation fraction can enhance forest productivity as scattered light penetrates further into the canopy^10^. The remaining two processes operate primarily via the climate, affecting vegetation indirectly. We evaluate the impact on TCRE of methane emissions from wetlands, and emissions of biogenic volatile organic compounds (BVOCs) from vegetation, which interact with other chemical species to influence atmospheric composition. The control and all experimental configurations of UKESM are summarised in Table 1 and implementation of the processes within the model is described in Methods.

**Table 1:** Summary of the eight configurations of UKESM used in this study

Process	ukesm-ctrl	ukesm-nodgvm	ukesm-nonlim	ukesm-df	ukesm-bvoc	ukesm-fire	ukesm-wch4	ukesm-allprocs
Nitrogen limitation	ON	ON	OFF	ON	ON	ON	ON	ON
Dynamic vegetation	ON	OFF	ON	ON	ON	ON	ON	ON
Diffuse fraction of Photosynthetically Active Radiation	0.4	0.4	0.4	Interactive	0.4	0.4	0.4	Interactive
Methane cycle	Concentration driven	Concentration driven	Concentration driven	Concentration driven	Concentration driven	Concentration driven	Emissions driven, interactive wetland emissions	Emissions driven, interactive wetland emissions
Fire-vegetation interactions	OFF	OFF	OFF	OFF	OFF	ON	OFF	ON
BVOCs	Prescribed	Prescribed	Prescribed	Prescribed	Interactive	Prescribed	Prescribed	Interactive

ON/OFF indicates that the process in column 1 is included/not included in the configuration, respectively. “Emissions driven” methane cycle indicates that the model calculates the methane concentration at the surface; when “concentration driven”, this is prescribed.

Adding such complex processes to an ESM is a slow process. In the seven years between the 5th and 6th phases of CMIP, the number of ESMs with nitrogen limitation rose from two to six of the 11 included in the definitive study on TCRE at CMIP6 by Arora et al. (2020) ^11^. Of these 11 ESMs, only five include fire-vegetation interactions, three permit dynamic vegetation distribution, three include interactive BVOC emissions, and three represent diffuse radiation impacts on vegetation. None simulates the emission of methane from wetlands interactively. Processes such as nitrogen limitation, fire and interactive wetland methane emissions are likely to become more prevalent in ESMs during the 7th phase of CMIP^12–17^. The ESM2025 project, for example, has funded the development of several ESMs to enhance their process representation: four ESMs now have, or are close to having, emissions-driven methane capability^16^, while others have extended their land carbon cycle with improved carbon-nitrogen coupling or more sophisticated fire processes^17^.

Although the experiments from which TCRE is derived are included in the core Fast Track phase of CMIP7^18^, it is unlikely that participating ESMs’ TCRE will be published before 2028. The 2025 Global Carbon Project^3^ concludes that from January 2026 onwards, emission of another 45 GtC of CO_2_, approximately four years’ worth at the 2025 emission rate, gives a 50% likelihood of exceeding 1.5 °C: we may be on the cusp of crossing that significant climate milestone when the CMIP7 ESMs’ TCREs become available.

The present study enhances our understanding of TCRE in several ways. First, we present the influence of each process on TCRE. We then partition the underlying causes between surface energy balance and carbon cycle processes, thereby improving our mechanistic understanding of the drivers of TCRE. We end with a summary of our main conclusions and a discussion about how we might expect TCRE to evolve in CMIP7 era models based on our results with UKESM.

## Results

### Process-dependence of the transient climate response to cumulative CO_2_ emissions

**Fig. 1:**
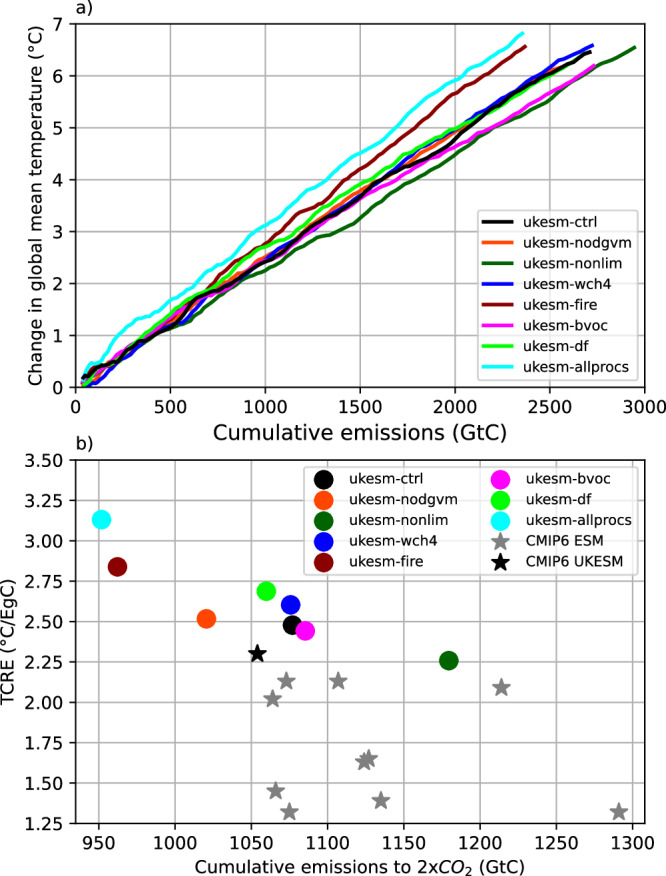
Temperature change, cumulative emissions and TCRE (transient climate response to cumulative CO_2_ emissions) of the UKESM process ensemble. **a** Change in global mean temperature vs cumulative emissions for the UKESM process ensemble, smoothed with a ten-year running mean. **b** TCRE at 2× CO_2_ vs cumulative emissions to 2× CO_2_ for the UKESM process ensemble (circles) and CMIP6 ESMs (stars, black star is UKESM1-0-LL).

The near-linear relationship between change in global mean temperature and cumulative carbon emissions is shown in Fig. 1a for the UKESM ‘process ensemble’ during the standard *1pctCO2* experiment, in which prescribed CO_2_ concentration rises at 1% annually from pre-industrial until quadrupling after 140 years (see Methods). The cumulative carbon emissions indicate how much CO_2_ can be emitted by fossil fuel burning to remain consistent with the CO_2_ concentration driving the simulation: the stronger (weaker) the land and ocean carbon sinks, the greater (lesser) the cumulative carbon emissions. The figure demonstrates a considerable spread in the coloured lines representing the six process ensemble members around the control configuration (‘*ukesm-ctrl’*, black line). The cyan line represents an additional configuration which contains all six processes, ‘*ukesm-allprocs’*, exhibiting the greatest warming as well as the lowest cumulative emissions of all configurations. Figure 1b shows the TCRE at 2× CO_2_ vs cumulative emissions to 2× CO_2_ for the UKESM process ensemble (coloured circles), and for 11 CMIP6 ESMs to provide context (stars). The CMIP6 version of UKESM (black star) had the highest TCRE (2.30 °C/EgC [Eg = exagram = 10^18^g]) of the 11 ESMs reported in Arora et al. (2020) ^11^: this is slightly exceeded by that of *ukesm-ctrl* (2.48 °C/EgC), mostly due to the impact of bias corrections and other improvements included in UKESM1.1^19^, allowing it to better recreate historical climate change (Supplementary Fig. 1). Our *ukesm-ctrl* configuration differs from the standard UKESM1.1 model documented in Mulcahy et al. (2023) ^19^ only through having prescribed preindustrial BVOC emissions.

The range in TCRE of the six single process-difference configurations is 0.58 °C/EgC (from 2.26 to 2.84 °C/EgC, Table 2). In the ‘*ukesm-nonlim’* and ‘*ukesm-nodgvm’* experimental configurations, a process present in *ukesm-ctrl* is disabled—nitrogen limitation of land carbon uptake, and dynamic vegetation, respectively. All other experimental configurations add a process absent from *ukesm-ctrl*. Expressing the change in TCRE as the impact of adding the process into the configuration in which it was absent, four of the processes increase TCRE, while two decrease it. Enabling fire-vegetation interactions (*‘ukesm-fire’*) increases TCRE by 14.6%, nitrogen limitation increases it by 9.7%, calculating the diffuse fraction of radiation from local conditions (*‘ukesm-df’*) increases TCRE by 8.5%, and interactive wetland methane emissions (*‘ukesm-wch4’*) increase it by 5.1%. Conversely, enabling dynamic vegetation reduces TCRE by 1.5%, and including interactive BVOC emissions (*‘ukesm-bvoc’*) reduces it by 1.4%.

**Table 2:** Diagnosed global quantities

Configuration	TCRE (°C/EgC)	Cumulative emissions to 2× CO_2_	TCR (°C)	Airborne fraction	*λ* (Wm^−2^ °C^−1^)
*ukesm-ctrl*	2.478	1077	2.669	0.564	0.735
*ukesm-nonlim*	2.258	1180	2.663	0.515	0.664
*ukesm-bvoc*	2.442	1085	2.651	0.559	0.793
*ukesm-nodgvm*	2.516	1021	2.569	0.595	0.752
*ukesm-wch4*	2.603	1076	2.801	0.564	0.709
*ukesm-df*	2.688	1060	2.849	0.573	0.733
*ukesm-fire*	2.839	962	2.732	0.631	0.674
*ukesm-allprocs*	3.131	952	2.980	0.638	0.776

Transient climate response to cumulative CO_2_ emissions (TCRE, °C/EgC); cumulative emissions to 2× CO_2_; transient climate response (TCR, °C), airborne fraction of cumulative emissions to 2× CO_2_; and diagnosed climate feedback parameter, *λ* (W m^−2^ °C^−1^), from the UKESM process ensemble.

When including *ukesm-allprocs*, containing all six processes, the UKESM process-driven range increases to 0.87 °C/EgC, comparable in magnitude to that of the 11-member CMIP6 ESM ensemble (0.98 °C/EgC), the members of which differ greatly in the physical and Earth system components they include and the couplings between them^11^. The configuration in which vegetation is not limited by nitrogen availability, *ukesm-nonlim*, has the lowest TCRE (2.26 °C/EgC) of the process ensemble, while *ukesm-fire* has the highest (2.84 °C/EgC) of the six single process-difference configurations.

**Fig. 2:**
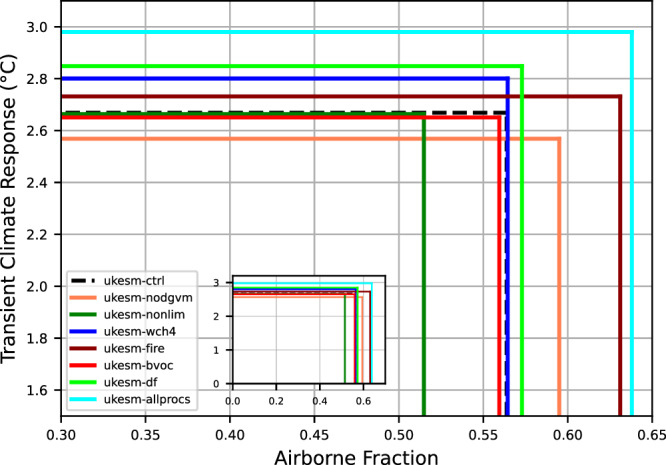
Transient climate response (TCR) vs airborne fraction of cumulative emissions to 2× CO_2_ of the UKESM process ensemble. The control configuration, *ukesm-ctrl*, is the dashed black line, and the experimental configurations are the solid, coloured lines. Transient climate response to cumulative CO_2_ emissions (TCRE) is proportional to the product of TCR and airborne fraction, and therefore to the area enclosed by the axes and the paired horizontal and vertical lines. For clearer separation of the individual configurations’ responses, the axes in the main plot are truncated, while the inset figure provides the full context, with each axis starting at zero.

The TCRE is proportional to the product of two metrics: the transient climate response (TCR)—the change in global mean temperature at the point of doubling of CO_2_ concentration during a *1pctCO2* experiment—and the airborne fraction (AF) of cumulative emissions to that point, the fraction of emitted carbon which remains in the atmosphere^20^. Figure 2 plots the TCR against the AF for each configuration, with the TCRE being proportional to the area enclosed by each pair of colour-coded lines. The contribution to TCRE of the experimental configuration’s perturbation to the carbon cycle (*x*-axis) and physical climate (*y*-axis) relative to *ukesm-ctrl* is evident from the figure and is recorded in Table S1 as a percentage. For example, the dark green horizontal line of *ukesm-nonlim* coincides almost exactly with the dashed black line of *ukesm-ctrl*, yet the dark green vertical line is the furthest to the left of the vertical black dashed line, indicating that the reduction in airborne fraction—and therefore the response of the carbon cycle rather than the physical climate—is almost entirely responsible (97.4%, Table S1) for the low TCRE of *ukesm-nonlim*. The airborne fraction is unchanged in *ukesm-wch4*, the configuration with interactive wetland methane emissions, so its TCRE deviates from that of *ukesm-ctrl* entirely (100%, Table S1) due to the change in TCR.

For *ukesm-nodgvm*, the small change in TCRE caused by disabling dynamic vegetation is the result of contrasting climate and carbon cycle responses, which almost cancel each other out. The increase in airborne fraction is second only to *ukesm-fire*, of the six single process-difference configurations, but this is offset by its low TCR, the lowest of all configurations. This leaves a small residual difference relative to *ukesm-ctrl*, corresponding to a 1.5% decrease in TCRE when dynamic vegetation capability is enabled.

In the next sections, we examine how the carbon cycle and physical responses in the process ensemble change the airborne fraction of emissions and TCR relative to *ukesm-ctrl*, to explain the contributions of each to TCRE seen in Fig. 2.

### Influence of the processes on the airborne fraction of carbon emissions

**Fig. 3:**
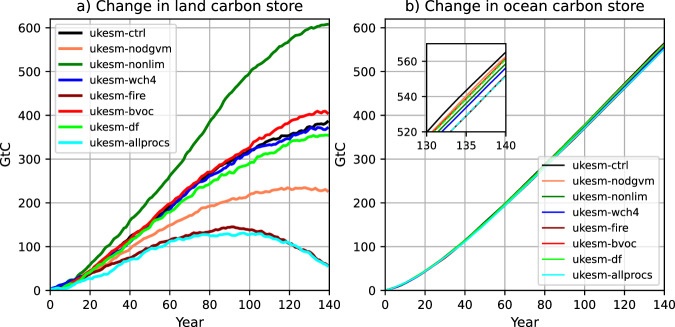
Change in land and ocean carbon stores of the UKESM process ensemble. Change in **a** land carbon store and **b** ocean carbon store in all configurations throughout the *1pctCO2* experiments. The black line is the control configuration, *ukesm-ctrl*, the coloured lines are the experimental configurations. The inset figure in panel **b** shows the final decade for clarity, in which *ukesm-df* and *ukesm-allprocs* appear as dashed lines to prevent obscuring *ukesm-bvoc* and *ukesm-fire* respectively.

The CO_2_ emissions consistent with the *1pctCO2* pathway driving the simulations (see Methods) can be diagnosed from the land and ocean carbon uptake and the changing CO_2_ concentration^21^: the amount by which the atmospheric carbon store increases divided by the diagnosed carbon emitted during the same period represents the airborne fraction of emissions, which is typically calculated cumulatively over the course of the simulation. Since the ocean carbon cycle scheme is common to all process configurations, ocean carbon uptake shows little configuration-dependence (Fig. 3b), changing only due to atmosphere-induced variations in sea-surface temperature, windspeed, and ocean circulation patterns.

Four of the six processes operate directly within the land carbon cycle and therefore influence net primary production explicitly, as well as indirectly through biophysical feedbacks to climate. Switching off nitrogen limitation of land carbon uptake strengthens considerably the cumulative net biosphere production (NBP), the land carbon sink. The map of cumulative (year 1 to year 70) NBP anomaly in *ukesm-nonlim* relative to *ukesm-ctrl* (Supplementary Fig. 2) shows that by the time CO_2_ has doubled, vegetation over much of the land surface has become nitrogen-limited. Alleviating the limitation in *ukesm-nonlim* permits an additional 227 GtC to be taken up by land over the full 140-year simulation (Fig. 3a), with 89 GtC sequestered to the point of 2× CO_2_, reducing the airborne fraction to 0.515 from the *ukesm-ctrl* value of 0.564.

Fixing the vegetation distribution in *ukesm-nodgvm* prevents the vegetation from adapting to both the changing climatic conditions and CO_2_ concentration; competition between plant functional types is height-dependent, with height being influenced by the PFT-specific sensitivity of photosynthesis to CO_2_, so the evolving PFT distribution in *ukesm-ctrl* is a function of both climate and CO_2_ concentration. The vegetation composition within *ukesm-nodgvm* is therefore less well adapted than that of *ukesm-ctrl*, and the land sink is weakened considerably (Fig. 3a): *ukesm-nodgvm* absorbs 151 GtC less than *ukesm-ctrl* over the 140-year simulation, and 54 GtC less by 2xCO_2_, increasing the airborne fraction from 0.564 to 0.595.

Introducing fire-vegetation interactions to the land carbon cycle in *ukesm-fire* represents a major development of the land carbon cycle, which directly limits carbon uptake through the removal of carbon from living biomass and from the two soil carbon stores fed by the plant litter flux^13^. As a result, the airborne fraction in *ukesm-fire* increases from 0.564 to 0.631, the highest of all configurations except *ukesm-allprocs*.

When the diffuse fraction of photosynthetically active radiation is calculated from local cloud cover and aerosol concentration, it can be greater or less than the constant fraction of 0.4 applied globally in *ukesm-ctrl*. This change in photosynthetic efficiency varies with time of day and location^22^, and results in a subtle change in vegetation composition relative to *ukesm-ctrl*. The net result is a minor reduction in land carbon uptake in *ukesm-df*, raising the AF from 0.564 to 0.573.

The remaining two configurations exert their impact on land carbon uptake entirely via their influence on climate: in *ukesm-wch4*, the AF is unchanged from *ukesm-ctrl* at 0.564, while *ukesm-bvoc*, which differs from *ukesm-ctrl* only through having interactive BVOC emissions, has a marginally lower AF of 0.559.

### Influence of the processes on transient climate response

The TCR ranges from 2.57 °C to 2.93 °C across the UKESM process ensemble (Table 2). Several processes impact TCR directly through changes in atmospheric composition. In *ukesm-bvoc*, BVOC emissions result in the formation of secondary organic aerosol, increasing the overall aerosol burden relative to *ukesm-ctrl*^22^: this induces marginal atmospheric cooling and a lower TCR. Conversely, interactive wetland methane emissions in *ukesm-wch4* increase the methane concentration by approximately 11% at 2× CO_2_ relative to *ukesm-ctrl*^22^, elevating TCR. Atmospheric composition in *ukesm-fire* is impacted by fire-induced emissions of several chemical species, including precursors which impact ozone and methane concentrations, and aerosols^13,23^ (see Methods) contributing to its high TCR relative to *ukesm-ctrl*.

The remaining configurations influence TCR only through their biophysical feedbacks to climate, two of which lead to the extremes of the TCR spread. TCR can be expressed approximately in terms of the global mean ocean heat uptake (OHU) and effective radiative forcing (ERF) at the point of doubled CO_2_ concentration in year 70 (OHU_70_ and ERF_70_), and the climate feedback parameter, *λ*, Eq. (1) (see Methods):

1 $${{\rm{TCR}}}\approx \frac{{{{\rm{ERF}}}}_{70}-{{{\rm{OHU}}}}_{70}}{\lambda }$$

We use the regression method of Andrews and Ringer^24^ to derive *λ* (Methods, Supplementary Fig. 3). For *ukesm-ctrl*, the regression yields *λ *= 0.735 W m^−2^ °C^−1^ with the process ensemble values ranging from 0.664 to 0.793 W m^−2^ °C^−1^ (Table 2). The 95% confidence intervals of the experimental configurations overlap with those of *ukesm-ctrl*, revealing their differences not to be statistically significant. This finding indicates that the spread in TCR is driven primarily by differences in the numerator of Eq. (1), (ERF_70_– OHU_70_), rather than by a significant shift in the global, temperature-dependent climate feedback parameter.

The first term in the numerator, ERF_70_, cannot be readily diagnosed in simulations with time-varying CO_2_. While dominated by the radiative forcing due to CO_2_ (Eq. (4), Methods), second-order contributions to ERF_70_ arise from feedbacks to climate due to biophysical changes which occur rapidly in response to the changing CO_2_, exerting a positive or negative increment to ERF_70_. As the vegetation in the experimental configurations deviates from that of *ukesm-ctrl* due to their process differences, the nature of these biophysical feedbacks varies across configurations. Maps of these differences between the experimental configurations and *ukesm-ctrl*, averaged over years 60–80, are shown in Supplementary Information, to illustrate how each contributes to ERF_70_: albedo (Supplementary Fig. 4), sensible and latent heat fluxes (Supplementary Figs. 5 and 6), and evaporation and transpiration (Supplementary Figs. 7 and 8).

The other term in the numerator, OHU_70_, is influenced by subtle shifts in ocean circulation patterns arising from changes in atmospheric temperature (Supplementary Fig. 9), precipitation (Supplementary Fig. 10), sea ice cover (Supplementary Fig. 11) and runoff from land.

### Low and high TCR extremes

The process with the lowest TCR is *ukesm-nodgvm* (2.57 °C). Disabling the dynamic vegetation scheme fixes the vegetation distribution, while in *ukesm-ctrl*, the global tree fraction increases from 0.33 to 0.37, replacing grasses and bare soil. This leads to a negative leaf area index (LAI) anomaly for *ukesm-nodgvm* over much of the land surface (Supplementary Fig. 12). While the evaporation and transpiration fluxes show a mixed response (Supplementary Figs. 7 and 8), the latent heat flux anomaly in *ukesm-nodgvm* is consistently negative over the land surface (Supplementary Fig. 6). Although reduced latent heat flux would typically exert a warming influence on land, the increased albedo of *ukesm-nodgvm* (Supplementary Fig. 4) shows that a greater fraction of incoming shortwave radiation is reflected. The net result is cooling over much of the land surface (Supplementary Fig. 12) and a reduced ERF_70_ compared to *ukesm-ctrl*, lowering TCR by 0.10 °C relative to *ukesm-ctrl*.

The highest TCR is observed in *ukesm-df*, in which photosynthesis is driven by the spatially varying diffuse fraction of photosynthetically active radiation (PAR). The climate feedback parameter, *λ*, is virtually unchanged by the interactive diffuse fraction scheme (0.733 W m^−2^ °C^−1^ compared to 0.735 W m^−2^ °C^−1^ for *ukesm-ctrl*), and the lack of strong, continental-scale shifts in factors affecting the surface energy balance (Supplementary Figs. 4–8), implies strongly that ERF_70_ is very close to that of *ukesm-ctrl*. Consequently, the key to the high TCR of *ukesm-df* appears to be OHU_70_. Supplementary Fig. 13 shows that cumulative ocean heat uptake in *ukesm-df* is the second lowest of all configurations, so its low OHU_70_ acts to increase the numerator. The Atlantic Meridional Overturning Circulation exhibits no significant weakening relative to *ukesm-ctrl* (Supplementary Fig. 14), so the suppressed OHU is likely due to changes in atmospheric circulation, which act on the ocean by increasing stratification and suppressing Southern Ocean Deep Water Formation. In the absence of any compensating factors which alter ERF_70_ or λ to offset the low OHU_70_, the TCR of *ukesm-df* is elevated relative to *ukesm-ctrl*, exceeded only by *ukesm-allprocs,* which contains all six processes. Note that some of the warming in *ukesm-df*, which gives it the highest TCR, is masked in Supplementary Fig. 9 by differences in the drift correction periods with respect to the *piControl* in the calculation of the TCR and the figure.

## Discussion

We have presented a systematic, factorial investigation quantifying the impact of individual Earth system processes on the Transient Climate Response to cumulative CO_2_ Emissions, using a highly sophisticated, fully coupled Earth System Model, UKESM1.1. The processes studied are a combination of long-established schemes like dynamic vegetation, which featured in some of the earliest ESMs^25^, and more recent developments which will be represented for the first time at CMIP7. Common to all processes is that they are not universally adopted by ESMs: nitrogen limitation, fire, interactive BVOC emissions and dynamic vegetation were included in some models at CMIP6, while interactive methane emissions will be present in around four models at CMIP7^14,16^. Our study therefore provides insight into inter-model differences in land carbon uptake, compatible emissions and the TCRE at CMIP6, and in CMIP7 to come.

In the literature, ESMs are commonly grouped into two cohorts: nitrogen-limited, and non-nitrogen limited^11^. Of the 11 CMIP6 ESMs reported in Arora et al. (2020)^11^, those including nitrogen limitation exhibited compatible emissions to 2× CO_2_ 6.6% lower, and TCRE 4.5% higher, than those which did not. We demonstrate explicitly that in UKESM1.1 accounting for the nitrogen demand of vegetation reduces the land carbon sink by suppressing the fertilisation effect of elevated CO_2_, increasing the airborne fraction of emitted carbon, thereby reducing cumulative compatible emissions to 2× CO_2_ by 9.6% and raising TCRE by 9.7%. Dynamic vegetation is often discussed as a major difference between ESMs^26,27^, but our findings show that its overall impact on TCRE (reducing it by 1.5%) is dwarfed by that of nitrogen limitation.

The process-induced changes in TCRE presented here are diagnosed from CO_2_ concentration-driven experiments. In CO_2_ emissions-driven mode, the large changes in airborne fraction observed in some configurations would equate to potentially significant changes in CO_2_-induced radiative forcing, widening further the spread in TCRE. For example, including fire-vegetation interactions increased markedly the airborne fraction: in emissions-driven mode, this would translate into a higher CO_2_ concentration relative to the control configuration for a given emissions scenario, driving up TCRE. Conversely, disabling nitrogen limitation, promoting land carbon uptake, would reduce atmospheric CO_2_ relative to *ukesm-ctrl*, delaying the point at which CO_2_ concentration doubles, thereby lowering TCRE. Hence, the process-induced spread in TCRE observed in our concentration-driven experiments may be somewhat conservative.

That CMIP6 comprises a mix of ESMs of varying complexity does not preclude calculating a CMIP6-mean TCRE value, but it does mean that interpreting it is difficult, and that estimating the remaining carbon budget to future warming targets is problematic. The Intergovernmental Panel on Climate Change (IPCC) usage of TCRE applies a correction term for “missing processes” when estimating a remaining carbon budget, but this is not fully consistent with the fact that some processes are partially included and partially missing. Hence, an ability to account for process-level impact on TCRE is needed to create a more internally consistent estimate of TCRE from CMIP ESMs.

We encourage other modelling groups to conduct similar studies using the factorial approach, to elucidate further the influence on TCRE of Earth system processes in isolation within ESMs—processes included in our study as well as others not yet represented within UKESM. In particular, the land surface components of ESMs are beginning to account for carbon loss from permafrost thaw^28^ and drying peatlands^29^, which could present a major negative perturbation to the land carbon sink^30,31^, while other processes such as phosphorus limitation^32^ and ozone damage to vegetation^33^ could also drive up the airborne fraction of emissions and raise the TCRE.

Our experiments indicate the potential direction of travel of TCRE in CMIP7: the introduction of four of our six processes increased TCRE by 9.5% on average, while two reduced it by an average of 1.5%. With the anticipated additional representation of interactive wetland methane emissions, fire and nitrogen limitation in CMIP7 ESMs^12–17^, our results suggest that, in the absence of any compensating cooling influence relative to their CMIP6 counterparts, an increase in TCRE is likely at CMIP7. The inverse relationship between TCRE and the remaining carbon budget to a future warming target such as 2 °C implies that if this is so, carbon budgets founded on TCRE estimates from CMIP7 ESMs would be lower than those derived from the CMIP6 cohort^34^, indicating the need for even starker emissions reductions in the coming decades.

## Methods

We use a control and six experimental variants of UKESM1.1, which is functionally similar to UKESM1-0-LL^35^, one of the 11 CMIP6 ESMs included in Arora et al. (2020) ^11^, but updated with bias corrections and bug fixes^19^. Starting from *ukesm-ctrl*, the experimental configurations were created by implementing all metadata and code changes necessary, which were then spun up with all forcing fixed at the pre-industrial (1850) level until the land carbon store was approximately in steady state. Once spun up, a pre-industrial control (‘*piControl*’) simulation was performed for each configuration, to provide a baseline against which to compare the transient simulation initialised from the same initial conditions. The transient simulations were the standard ‘*1pctCO2’* experiments in which atmospheric CO_2_ concentration is incremented at 1%/year for 140 years, during which time it rises from pre-industrial to 4x the pre-industrial level. We briefly describe *ukesm-ctrl* and the experimental configurations below; they are summarised in Table 1. A full description of UKESM1.1 is provided at the end of this section.

### ukesm-ctrl

Our control configuration includes two of the processes under consideration: nitrogen limitation of land carbon uptake and dynamic vegetation. Atmospheric methane concentration at the surface is prescribed. Fire is not represented. Emissions of BVOCs are prescribed at pre-industrial rates. The diffuse fraction of photosynthetically active radiation is fixed globally at 0.4.

### ukesm-nodgvm

Identical to *ukesm-ctrl* except competition between plant functional types has been disabled, so the spatial distribution of the vegetation is fixed throughout, though the canopy height and leaf area index change in accordance with the seasonal cycle, and as the climate changes.

### ukesm-nonlim

In this configuration, the nutrient demand of the vegetation is assumed to be met, and all carbon from net primary productivity is assimilated, with none lost to exudates; the nitrogen and carbon cycles are effectively decoupled.

### ukesm-wch4

This configuration uses a prognostic, emissions-driven methane scheme^14^ in which the surface CH_4_ concentration is not constrained to a climatology but is incremented by the methane flux from wetlands, and from prescribed pre-industrial anthropogenic sources and biogenic sources such as termites. In all other configurations, the surface methane concentration is prescribed (referred to as “concentration-driven” in Table 1).

### ukesm-bvoc

The fluxes of BVOCs are calculated every timestep and added as source terms to UKCA, where they take part in reactions of many chemical species and aerosols^23^, leading to the formation of Secondary Organic Aerosol, impacting CH_4_ and ozone concentration. The magnitude of the BVOC emission fluxes is dependent on the evolving vegetation composition.

### ukesm-df

The fraction of photosynthetically active radiation which is diffuse rather than direct is calculated every timestep in every gridbox, changing with local aerosol concentration and cloud cover. This is provided to the photosynthesis calculations rather than the value of 0.4 as used universally in *ukesm-ctrl* and all other configurations.

### ukesm-fire

Fire-vegetation interactions are handled by the INFERNO fire scheme^36,37^. Burnt area extent is determined by INFERNO as a function of soil moisture and vegetation cover, with the ignition rate determined from live lightning strikes simulated within the atmosphere model, as well as from human-induced ignitions, which are a function of the population density, provided externally at a pre-industrial level. Carbon lost due to combustion from above-ground biomass and from the two soil carbon pools containing decomposable and resistant plant material provides a diagnosed fire-induced CO_2_ emission flux; this does not impact atmospheric CO_2_ concentration, which is prescribed. Combustion-induced emissions of numerous chemical species and aerosols are coupled to the UKCA chemistry and aerosols scheme^13,23^: CO, NOx, C_2_H_6_, C_3_H_8_, HCHO, MeCHO, Me_2_CO, NH_3_, dimethyl sulphide, organic carbon and black carbon. Emissions of methane from fire are diagnostic only, since *ukesm-fire* does not include the interactive methane cycle^14^ employed in *ukesm-wch4*.

### ukesm-allprocs

An eighth configuration to illustrate the impact on TCRE of including all six processes.

#### Diagnosis of TCRE

TCRE is diagnosed from the idealised *1pctCO2* experiment in which CO_2_ concentration increases at 1%/year, and all non-CO_2_ forcing is maintained at the pre-industrial (1850) level. It is defined as the change in global mean temperature at the point of doubling of atmospheric CO_2_, divided by the carbon content of the cumulative CO_2_ emissions to that point, expressed in units of °C/EgC, or °C/1000 PgC (PgC=petagrams (10^15^g) of carbon). Increasing at 1% per year, CO_2_ concentration doubles in year 70, so we calculate the change in global mean temperature as the difference averaged over years 60–80 of the *1pctCO2* simulation relative to the average of the first 20 years of the *piControl* simulation run in parallel with the *1pctCO2*. Note that we calculate the temperature anomaly in years 60 to 80, centred on year 70, to be consistent with the CO_2_ doubling, rather than years 61–80 typically used in the calculation of TCR. Carbon emissions compatible with the prescribed CO_2_ concentration were calculated from the net land and ocean carbon uptake and the change in atmospheric CO_2_ burden using Eq. 2 of Liddicoat et al. (2021) ^21^, with CE_2×CO2_ being the cumulative total to year 70.

#### Derivation of the effective climate feedback parameter and TCR expression

The processes under investigation are all land-based but exert a forcing on the climate directly and indirectly in various ways: through changes in atmospheric composition, fluxes of heat and moisture to the atmosphere, and changes to the surface energy budget, thereby influencing atmosphere–ocean exchange. From the energy budget framework, the change in the net top-of-the-atmosphere energy flux (ΔN) in W m^−2^, is equal to the difference between the forcing, Δ*F*, due to a perturbation in the system, and the planet’s thermal response to that forcing, *R*(*t*):

2 $$\Delta N(t)=\Delta F(t)-R(t)$$

where positive is downwards, into the Earth system (adapted from Forster et al. (2021) ^38^). Assuming that the response, *R*(*t*), is proportional to the change in global mean temperature *T*(*t*) in °C, so that *R* = *λ*Δ*T*, where *λ* is the climate feedback parameter in W m^−2^ °C^−1^, results in the following expression:

3 $$\Delta N(t)=\Delta F(t)-\lambda \Delta T(t)$$

In the *1pctCO2* experiments, the perturbation applied to the system is the 1% increment in CO_2_ concentration applied annually, starting at pre-industrial concentration. Δ*F*(*t*) is the time-varying effective radiative forcing (ERF) caused by this perturbation, relative to the pre-industrial. Direct radiative forcing due to CO_2_ dominates the ERF: the stomatal response of the vegetation to changing CO_2_ impacts evaporation and transpiration, and therefore latent and sensible heat fluxes, providing minor positive and negative increments to the overall forcing, but this is of secondary importance^39^. Therefore, we set Δ*F*(*t*) to ERF_CO2_ (W m^−2^) using the logarithmic relationship calibrated to UKESM1-0-LL^40^:

4 $$\left.{{\mathrm{ERF}}}_{{\mathrm{CO}}2}(t)=5.34\,{\mathrm{ln}}(C(t)/{C}_{0})\right)$$

where *C*(*t*) is the CO_2_ concentration in year *t*, and *C*_0_ is the initial (1850) CO_2_ concentration. Substituting into Eq. (3) yields:

5 $$\Delta N(t)={{\mathrm{ERF}}}_{{\mathrm{CO}}2}(t)-\lambda \Delta T(t)$$

Plotting a linear regression of Δ*N*(*t*) − ERF_CO2_(*t*) against Δ*T*(*t*) yields a straight line whose gradient is −*λ* (see Supplementary Fig. 3): a positive *λ* implies a stabilising influence on climate, with increasing *λ* acting to reduce the TCR^24^.

When CO_2_ has doubled, in year 70, the change in global mean temperature is equal to the transient climate response, Δ*T* = TCR, and Δ*F* = ERF_70_ is the total effective radiative forcing at 2× CO_2_; substituting these into Eq. (5) yields

6 $$\lambda {\mathrm{\cdot TCR}}={{\mathrm{ERF}}}_{70-}\Delta N$$

The vast majority of heat entering the Earth system is taken up by the ocean, so to first order, Δ*N* can be approximated as the ocean heat uptake (OHU) in year 70, OHU_70_ in W m^−2^ (note that we show a timeseries of cumulative OHU in Supplementary Fig. 13, which illustrates the time history of ocean heat uptake more clearly than the instantaneous OHU rates). Substitution into Eq. (6) yields the expression for TCR:

7 $${{\rm{TCR}}}\approx \frac{{{{\rm{ERF}}}}_{70}-{{{\rm{OHU}}}}_{70}}{\lambda }$$

Examination of the terms on the RHS of Eq. (7) helps to explain the magnitude of TCR across the different configurations.

Note that we assume the same calibrated logarithmic formula for ERF_CO2_ (Eq. (4)) applies equally to all configurations of UKESM. This may not be the case if any of the additional interactive processes significantly impact the adjustment processes that are included in the ERF definition. The uncertainty in ERF is therefore subsumed into our *λ*, but since the ERF is strongly dominated by the direct CO_2_-induced radiative forcing, we would expect the associated uncertainty in *λ* to be small.

#### Description of UKESM1.1

The following is a description of the control configuration of UKESM1.1 used in the present study.

The atmosphere component of the model is vn12.0 of the Met Office’s Unified Model, which is coupled to vn6.1 of the JULES land surface model^41,42^.

The land surface is populated by 13 plant functional types (PFTs) ^43^, of which nine are natural: three broadleaf tree types (evergreen-tropical, evergreen-temperate, and deciduous), two needleleaf trees (evergreen, deciduous), two shrubs (evergreen, deciduous) and two grasses (C3 and C4 photosynthetic pathways). The four agricultural types are represented by C3 and C4 variants of crop and pasture PFTs, which are phenologically similar to natural grasses, but are constrained to grow only within a fraction of the gridbox assigned to agricultural land use. In all experiments in this study, land use was fixed at the pre-industrial distribution. Vegetation composition is free to evolve as trees, shrubs and grasses compete for available space within a gridbox; competition between PFTs is governed by height advantage^43^.

Litter from vegetation is passed to decomposable and resistant plant material (DPM and RPM) soil carbon pools, from which carbon passes into the biomass and humified organic matter pools. Each of the four soil carbon pools has its own decomposition rate, leading to a decomposition CO_2_ flux to the atmosphere which is diagnostic only in these prescribed-CO_2_ experiments. The four soil carbon pools are matched by nitrogen equivalents, with an additional inorganic nitrogen pool to which the vegetation has access to satisfy its nutrient demand, and to which nitrogen is added from bacterial nitrogen fixation and atmospheric deposition. When the inorganic nitrogen pool is insufficient to meet the nitrogenous nutrient demand of a PFT, its net primary production (NPP) is downregulated until consistent with the available supply; non-assimilable carbon due to excess NPP constitutes an exudate flux.

In the standard configuration of UKESM1.1, emissions of BVOCs calculated within JULES from the evolving vegetation distribution and environmental conditions are passed to the UKCA chemistry and aerosols module, influencing atmospheric composition through interactions with other reactive species: *ukesm-bvoc* is configured in this way. In *ukesm-ctrl* and all other configurations, BVOC emissions are prescribed at pre-industrial rates. The fraction of photosynthetically active radiation which is diffuse is fixed at 0.4 in all gridboxes. Fire-vegetation interactions are not represented.

Above the surface, methane is carried as a 3D tracer: the concentration of methane within an atmosphere gridbox is influenced by fluxes into and out of it due to dynamical physical processes, and by its inclusion in chemical reactions simulated by the UKCA (UK Chemistry and Aerosols) model^23^. At the surface, methane is prescribed to prevent drift. Wetland extent changes in response to a changing climate; the emission flux of methane from wetlands is a function of NPP and is diagnostic only.

The physical ocean model is provided by vn3.6 of the NEMO ocean model^44^, coupled to vn2.0 of the Medusa ocean biogeochemistry model^45^. The flux of CO_2_ from the atmosphere to the ocean is calculated every ocean model timestep as a function of the atmospheric CO_2_ concentration, wind speed and the dissolved inorganic carbon content of the surface ocean.

## Supplementary information

Supplementary Information

Peer Review file

## Data Availability

The data^46^ from which the figures in the main text were created are published at 10.6084/m9.figshare.31182292.v1.

## Code availability

No specialist code is required to generate the plots included in the main paper or the Supplementary Information, which were created by standard Python routines.
