# Supplementary material for: Role of Earth system processes in the relationship between climate change and cumulative carbon emissions
Source: Nat Commun. 2026 May 19;17:6722. doi: 10.1038/s41467-026-72930-7 (PMC13385852; doi:10.1038/s41467-026-72930-7)

Supplementary Information for

Role of Earth system processes in the relationship between climate change and cumulative carbon emissions

Spencer K. Liddicoat*, Timothy Andrews, Chris D. Jones, Lina M. Mercado, Mark A. Ringer, Eddy Robertson, Stephen Sitch, Andy Wiltshire

*Corresponding author: spencer.liddicoat@metoffice.gov.uk

Supplementary Table 1: Contributions of the process-driven changes in Airborne Fraction (AF) and Transient Climate Response (TCR) relative to *ukesm-ctrl* to the change in TCRE, expressed as a percentage of the change in TCRE. In the case of *ukesm-nodgvm*, the small TCRE increase relative to *ukesm-ctrl* is the result of relatively large opposing differences in AF and TCR.

Configuration	TCRE (°C/EgC)	Change in TCRE relative to *ukesm-ctrl* (°C/EgC)	Contribution (%) to change in TCRE from change in:	
			AF	TCR
ukesm-ctrl	2.478	-	-	-
ukesm-nonlim	2.258	-0.220	97.4	2.6
ukesm-nodgvm	2.516	+0.038	342.7	-242.7
ukesm-wch4	2.603	+0.125	0.0	100.0
ukesm-df	2.688	+0.210	19.5	80.5
ukesm-fire	2.839	+0.361	82.8	17.2
ukesm-bvoc	2.442	-0.036	56.4	43.6
ukesm-allprocs	3.131	+0.653	52.8	47.2

Supplementary Figure 1: Global mean 1.5m air temperature anomaly relative to 1961-1990 average. Emissions-driven historical simulation with *ukesm-allprocs* configuration (red curve) and UKESM1-0-LL (blue curve), with observations-based values from HadCRUT5.0.2.0 (black curve, 2.5% to 97.5% confidence interval is shaded).

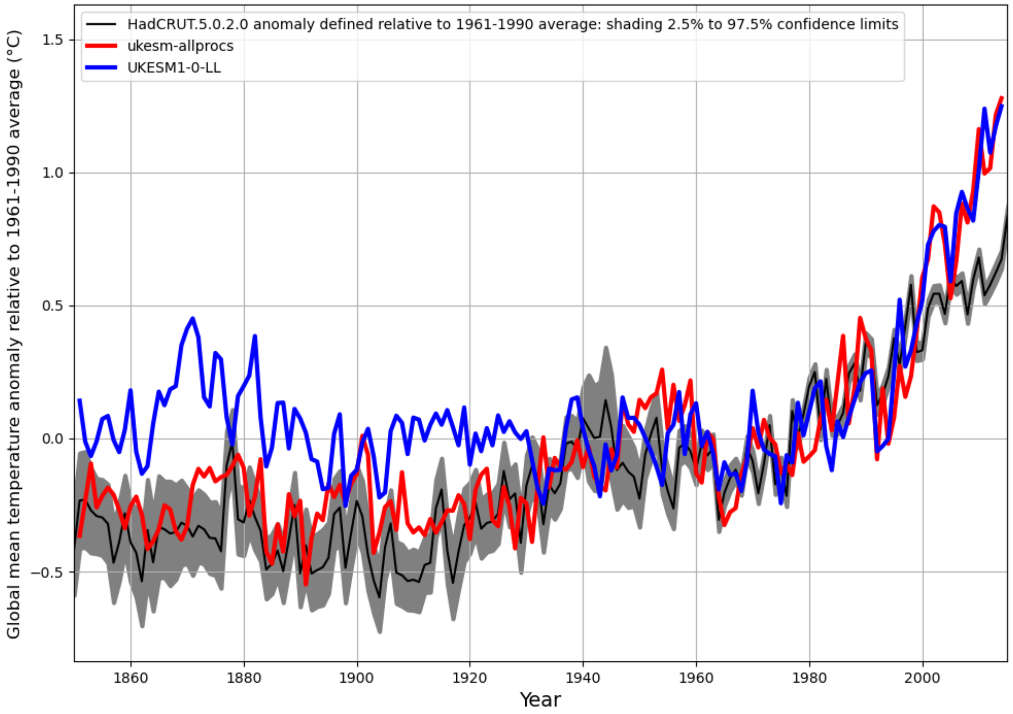

Supplementary Figure 2: Cumulative net biosphere productivity (NBP) anomaly (kg m^{-2} yr^{-1}) of each configuration relative to *ukesm-ctrl* at 2xCO_2 in the fully coupled *1pctCO2* experiment, from 1850 to 1920, drift-corrected by subtracting the equivalent of the configuration-specific *piControl* simulation.

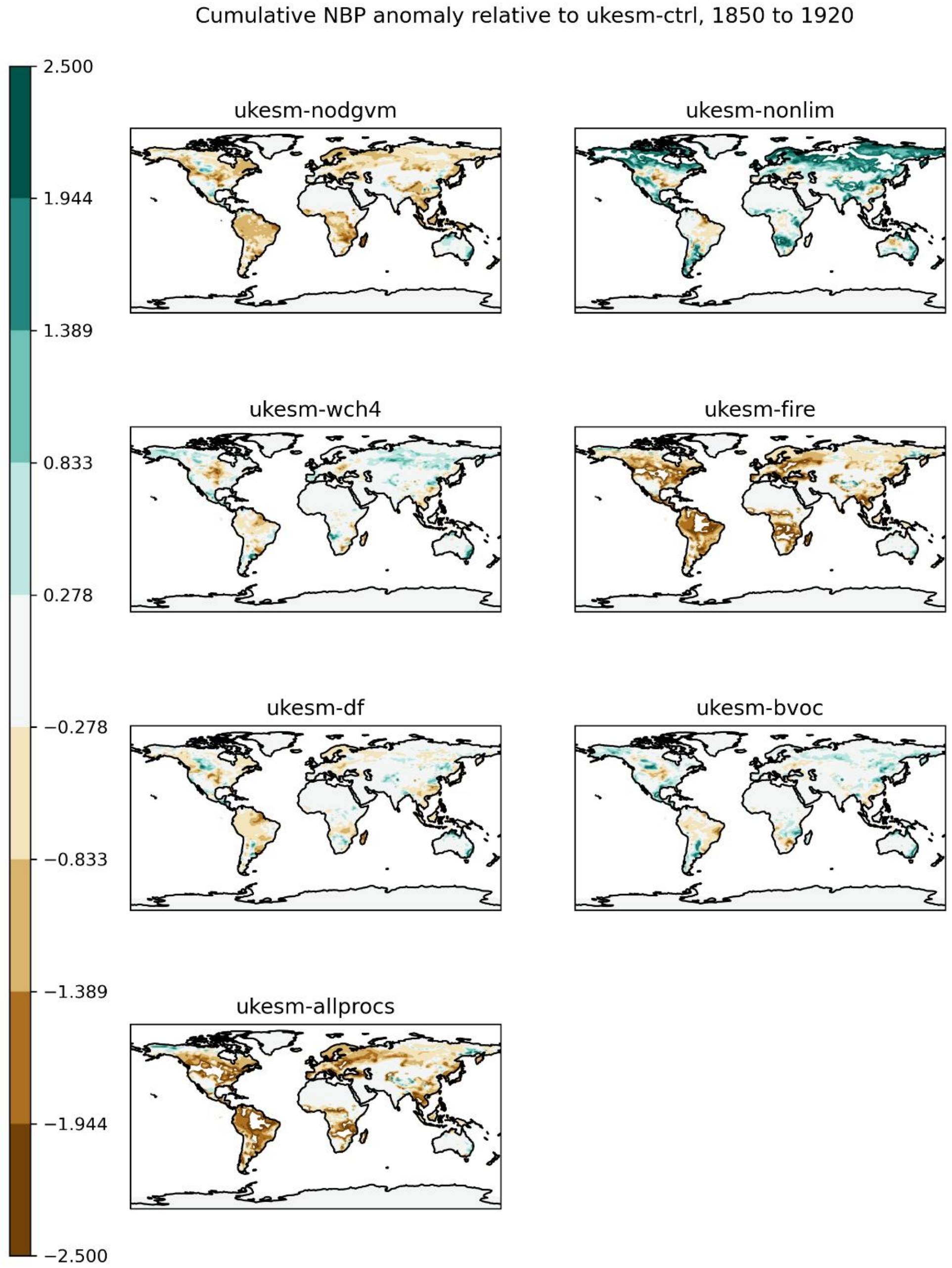

Supplementary Figure 3: Regression of [net top-of-the-atmosphere radiation flux – Effective Radiative Forcing due to CO_2] against change in global mean surface air temperature for diagnosing the climate feedback parameter, λ.

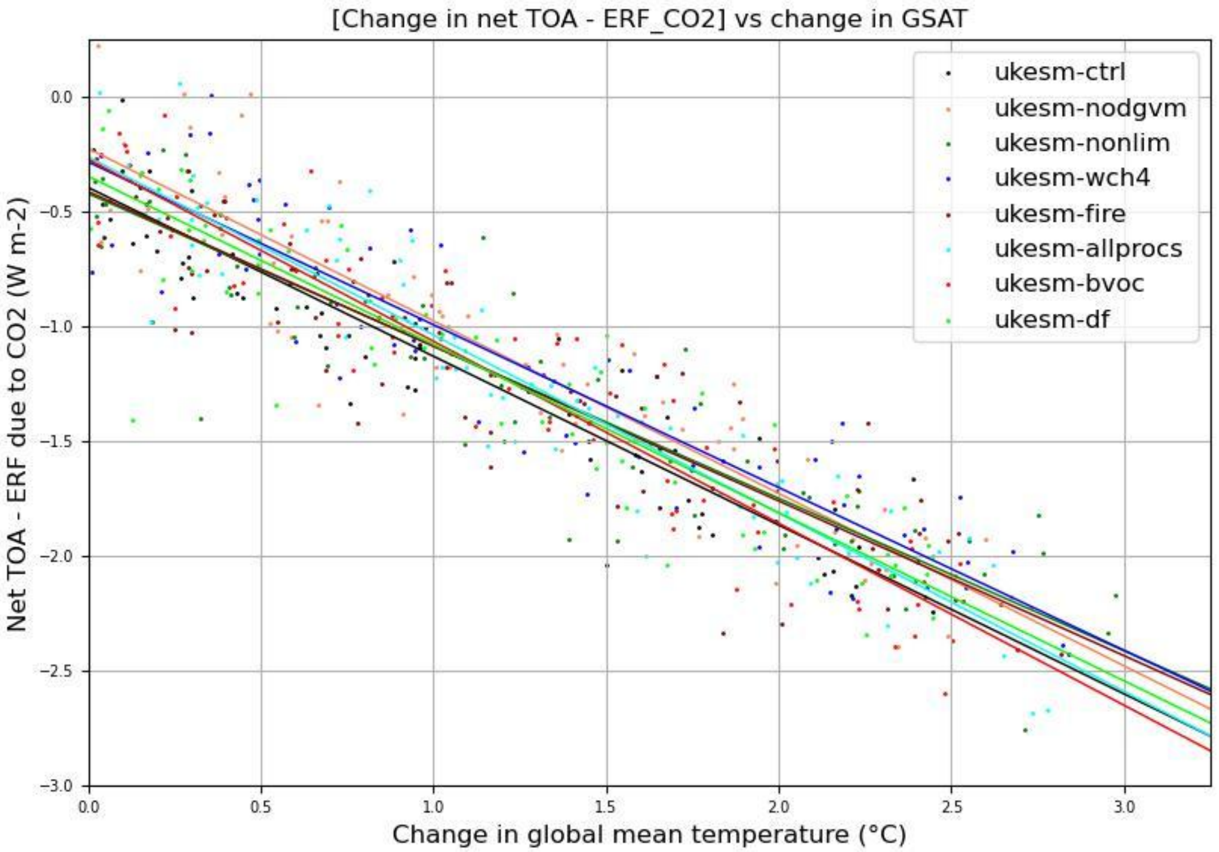

Supplementary Figure 4: Albedo anomaly of each configuration relative to *ukesm-ctrl* at 2xCO_2 in the fully coupled *1pctCO2* experiment, averaged over years 60 to 80 (1910 to 1930) and drift-corrected by subtracting the average of years 60 to 80 of the configuration-specific *piControl* simulation. Blues indicates higher albedo relative to *ukesm-ctrl* (implying a cooling effect), reds lower than *ukesm-ctrl* (warming).

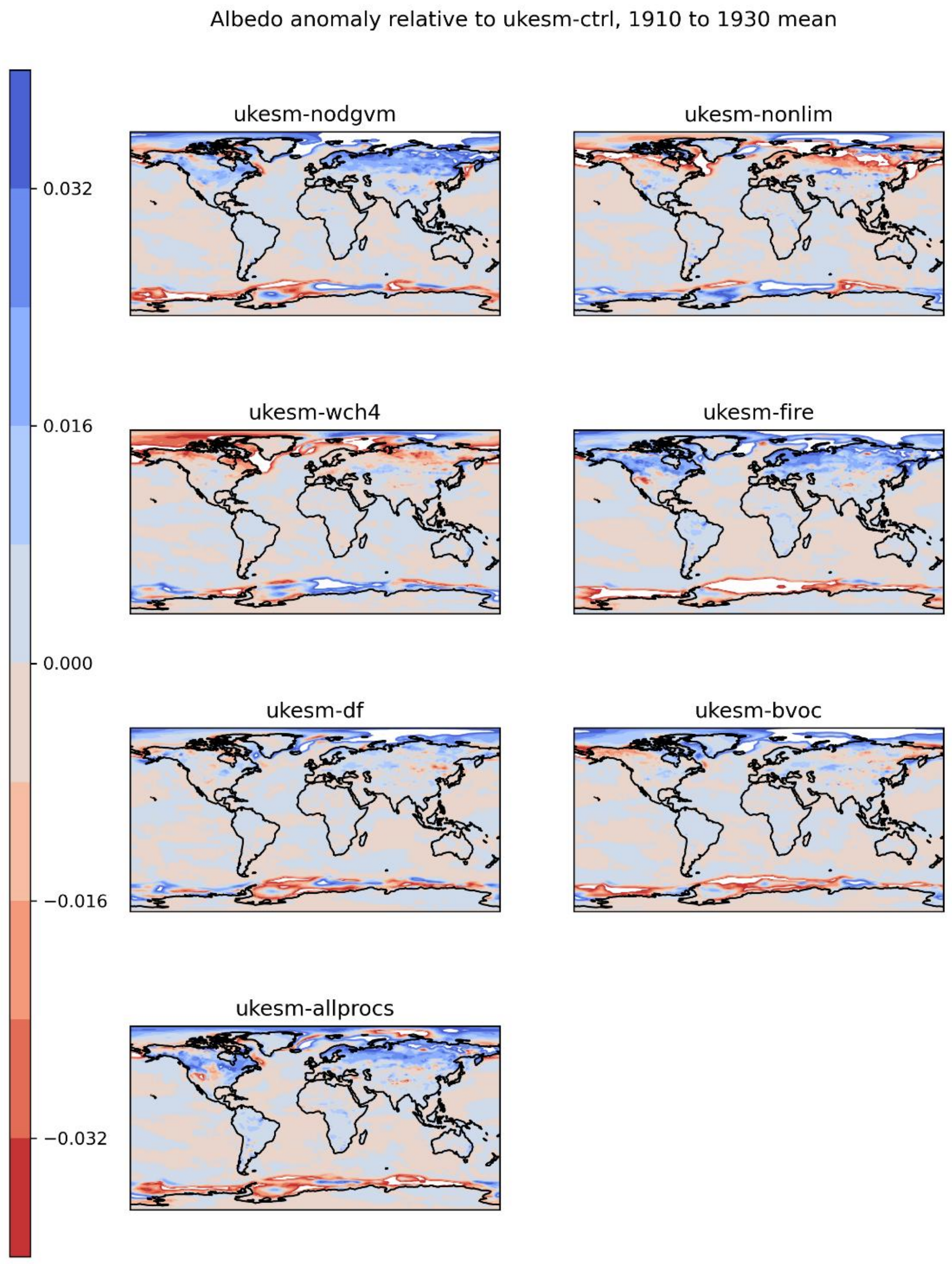

Supplementary Figure 5: Sensible heat flux anomaly (Wm^{-2}) of each configuration relative to *ukesm-ctrl* at $2xCO_2$ in the fully coupled *1pctCO2* experiment, averaged over years 60 to 80 (1910 to 1930) and drift-corrected by subtracting the average of years 60 to 80 of the configuration-specific *piControl* simulation.

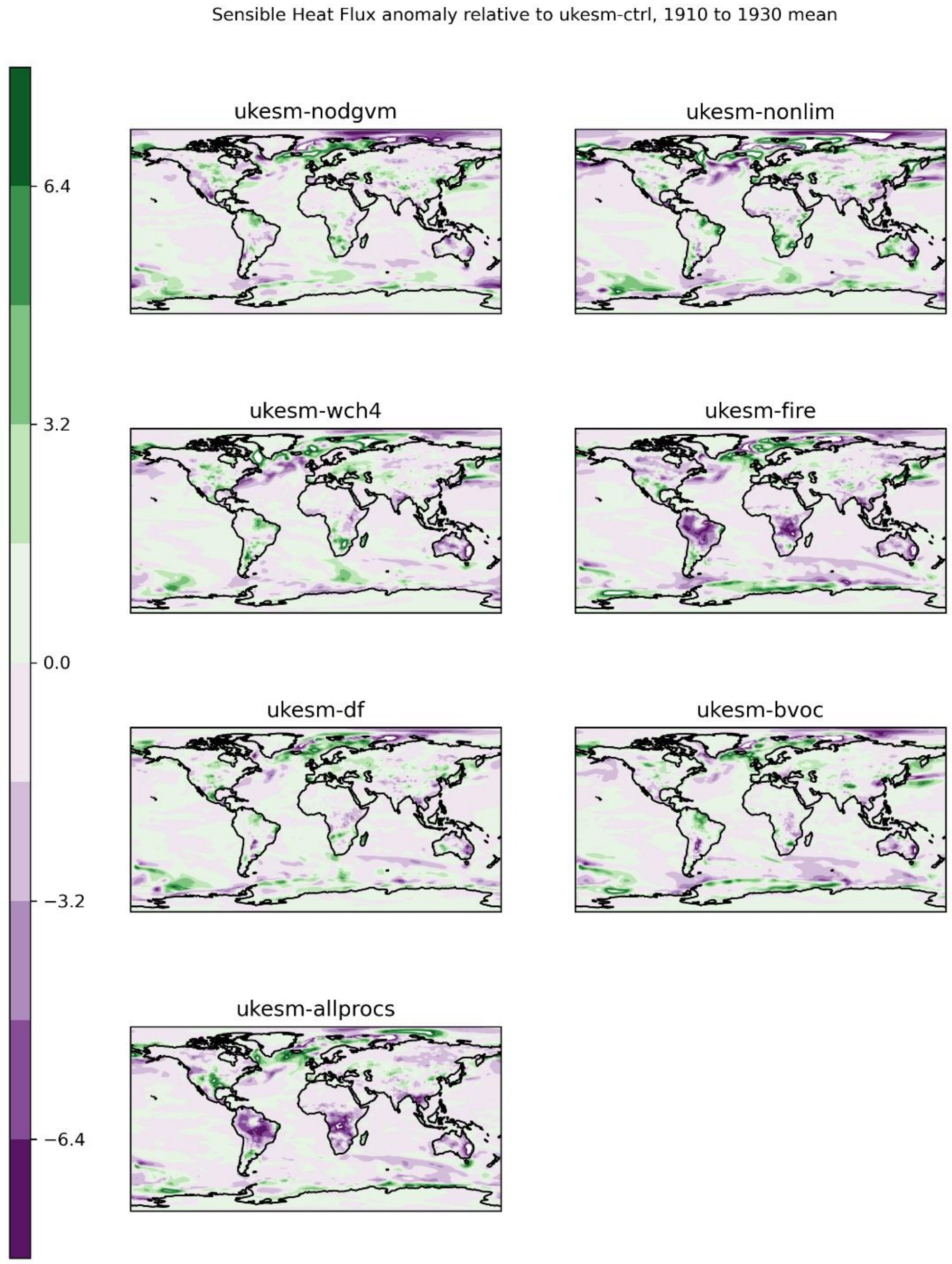

Supplementary Figure 6: Latent heat flux anomaly (Wm^{-2}) of each configuration relative to *ukesm-ctrl* at $2xCO_2$ in the fully coupled *1pctCO2* experiment, averaged over years 60 to 80 (1910 to 1930) and drift-corrected by subtracting the average of years 60 to 80 of the configuration-specific *piControl* simulation.

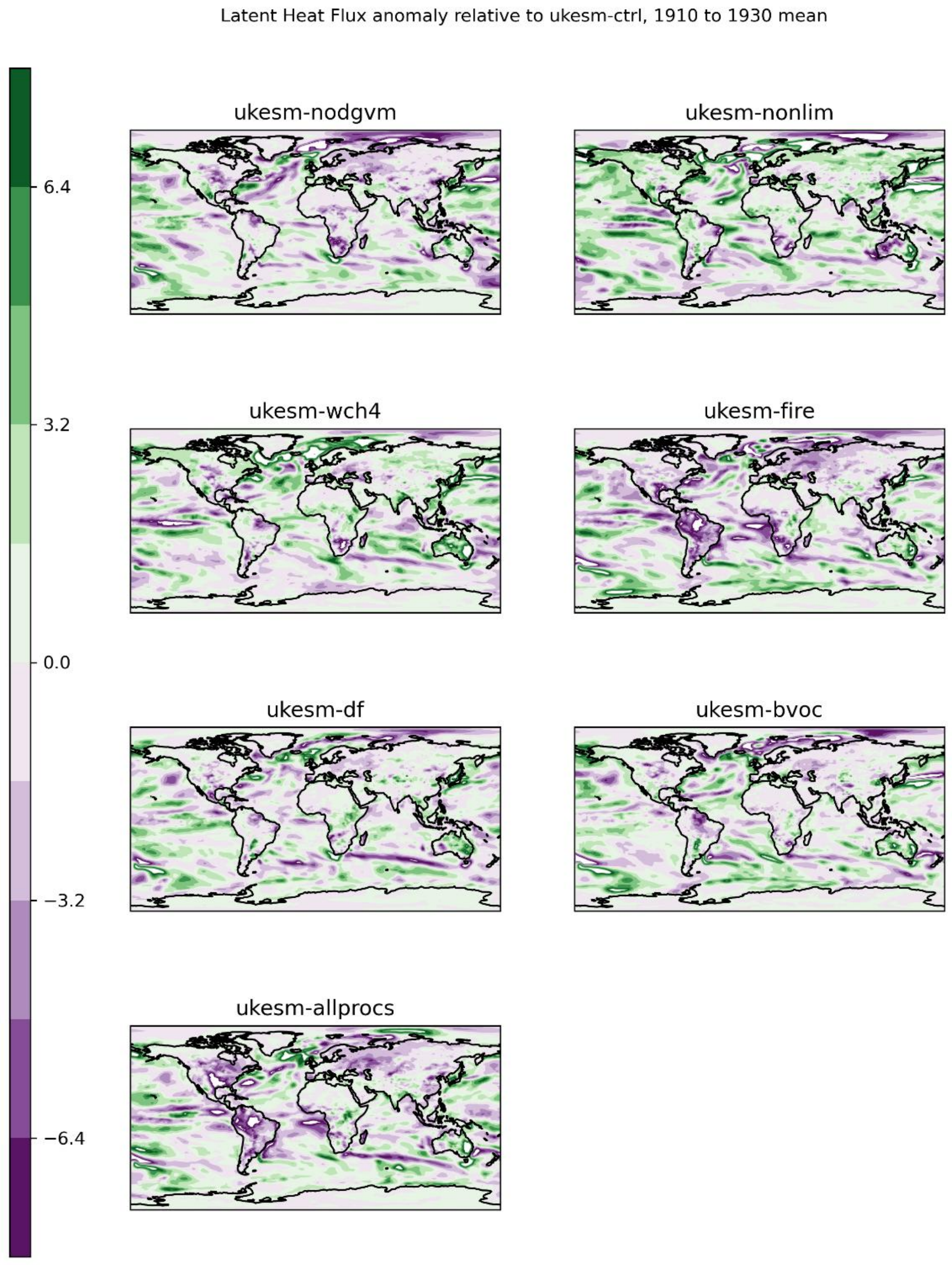

Supplementary Figure 7: Evaporation anomaly (kg m^{-2} s-1) of each configuration relative to *ukesm-ctrl* at 2xCO_2 in the fully coupled *1pctCO2* experiment, averaged over years 60 to 80 (1910 to 1930) and drift-corrected by subtracting the average of years 60 to 80 of the configuration-specific *piControl* simulation.

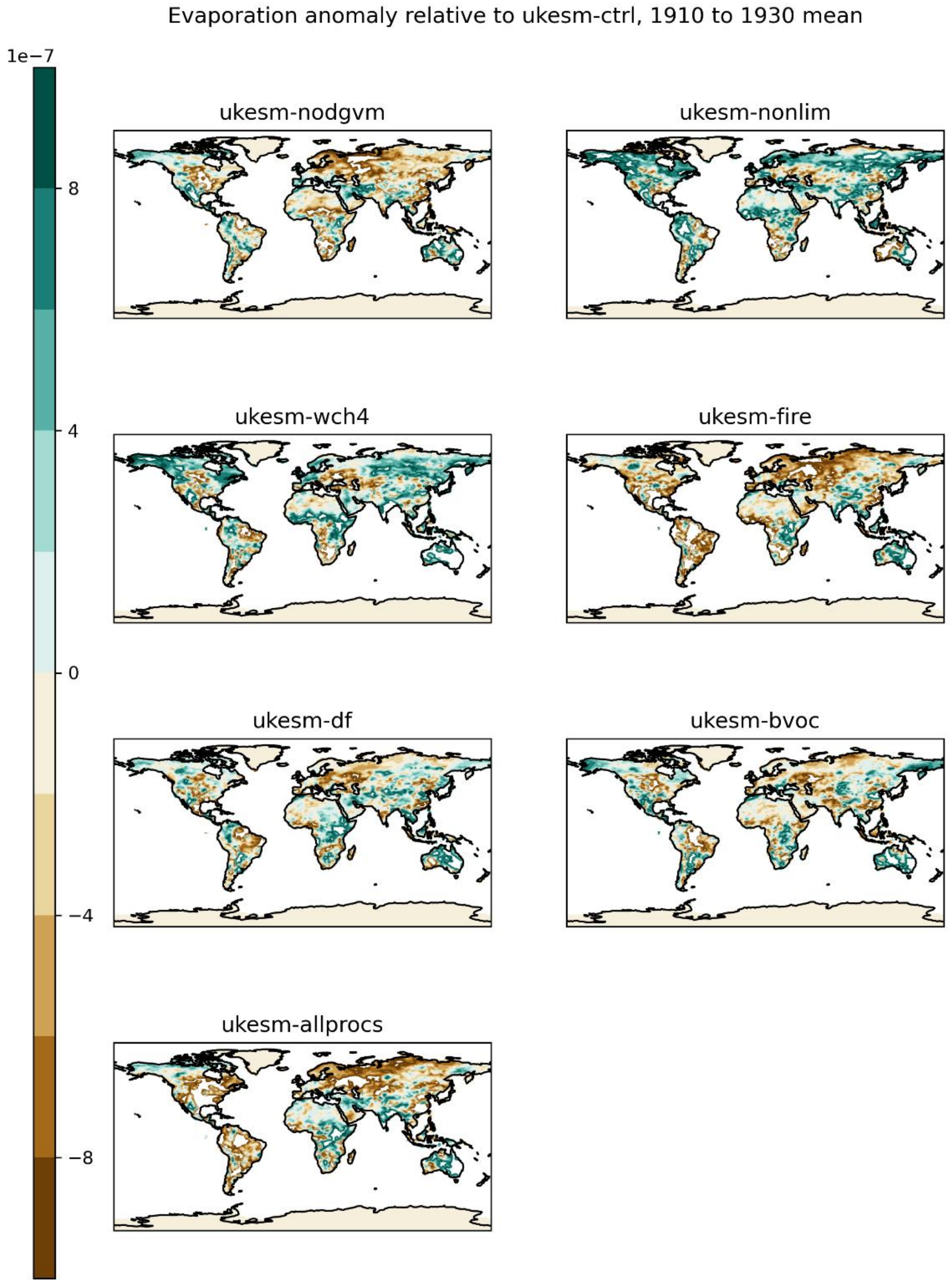

Supplementary Figure 8: Transpiration anomaly (kg m^{-2} s-1) of each configuration relative to *ukesm-ctrl* at 2xCO_2 in the fully coupled *1pctCO2* experiment, averaged over years 60 to 80 (1910 to 1930) and drift-corrected by subtracting the average of years 60 to 80 of the configuration-specific *piControl* simulation.

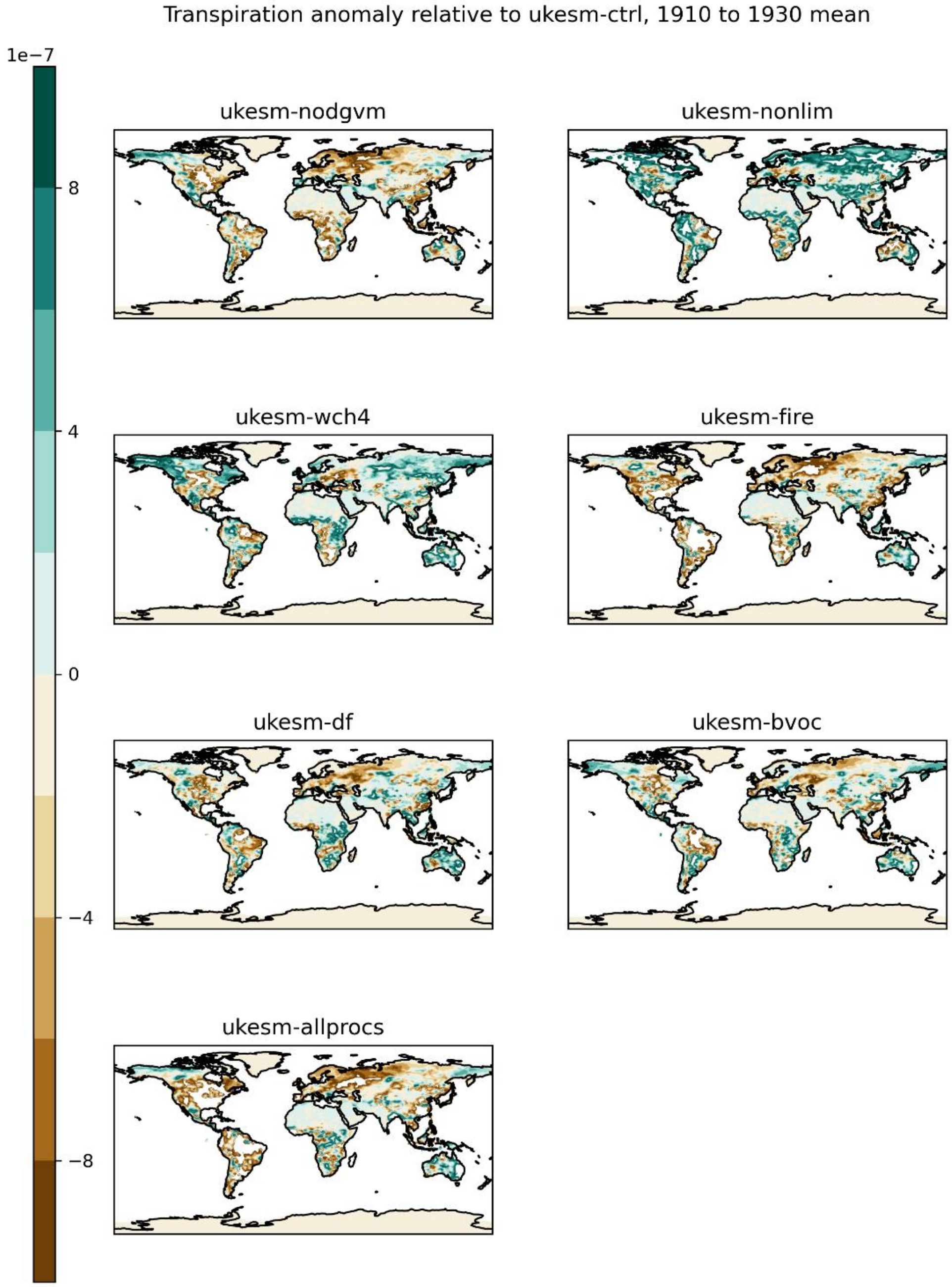

Supplementary Figure 9: 1.5m temperature anomaly (°C) of each configuration relative to *ukesm-ctrl* at 2xCO_2 in the fully coupled *1pctCO2* experiment, averaged over years 60 to 80 (1910 to 1930) and drift-corrected by subtracting the average of years 60 to 80 of the configuration-specific *piControl* simulation.

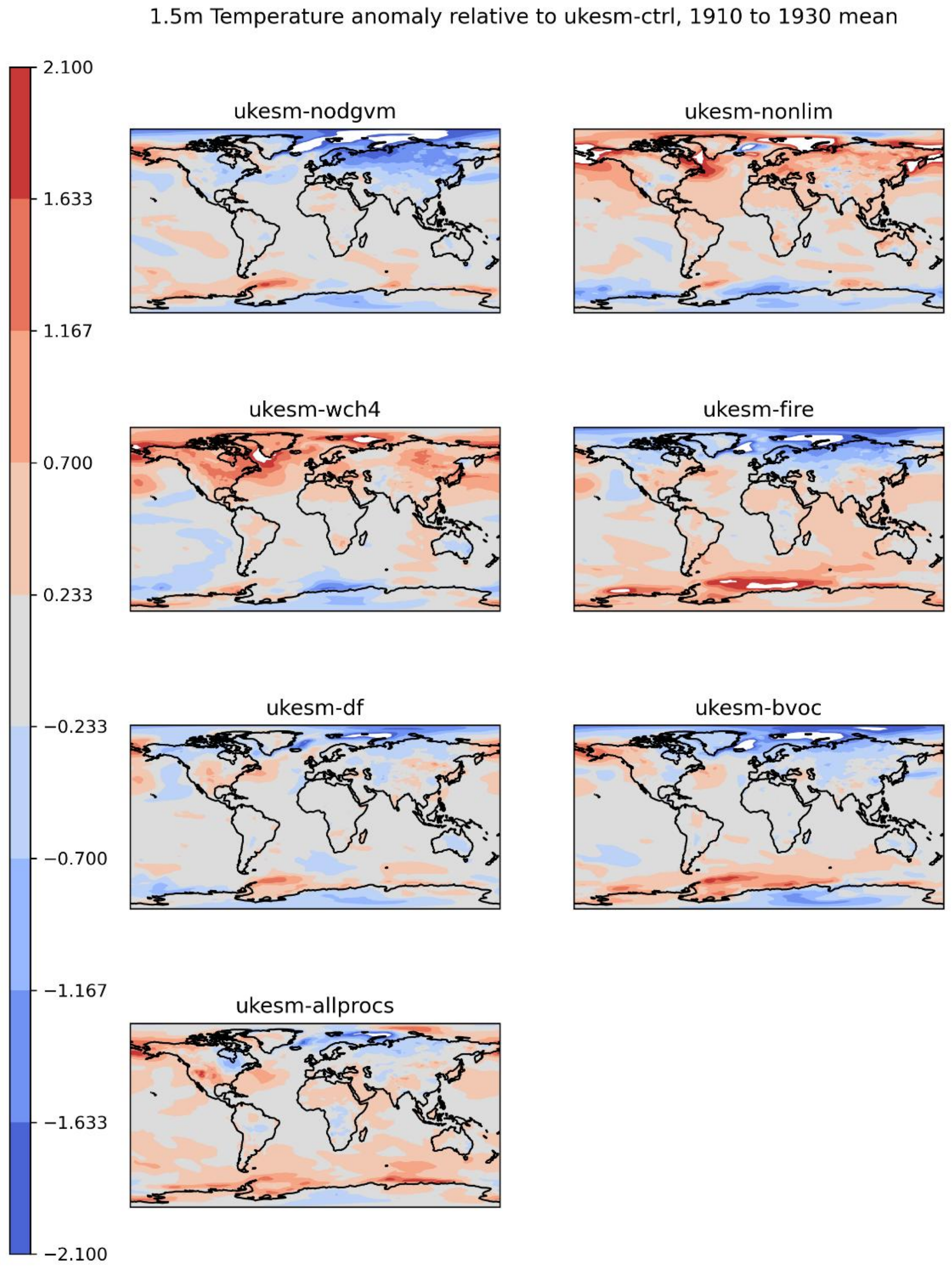

Supplementary Figure 10: Precipitation anomaly (mm day^{-1}) of each configuration relative to *ukesm-ctrl* at 2xCO_2 in the fully coupled *1pctCO2* experiment, averaged over years 60 to 80 (1910 to 1930) and drift-corrected by subtracting the average of years 60 to 80 of the configuration-specific *piControl* simulation.

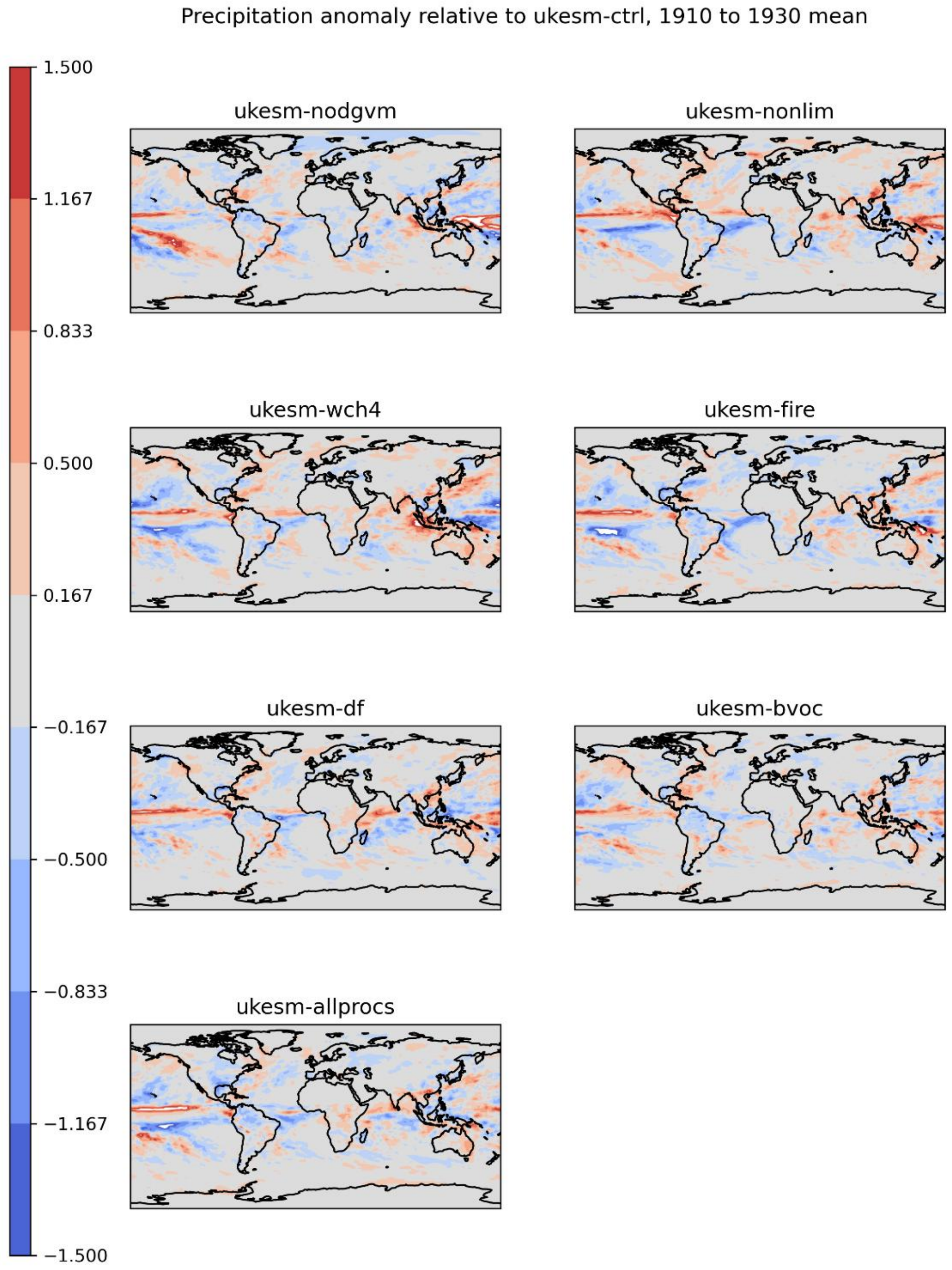

Supplementary Figure 11: Sea ice fraction anomaly of each configuration relative to *ukesm-ctrl* at 2xCO_2 in the fully coupled *1pctCO2* experiment, averaged over years 60 to 80 (1910 to 1930) and drift-corrected by subtracting the average of years 60 to 80 of the configuration-specific *piControl* simulation.

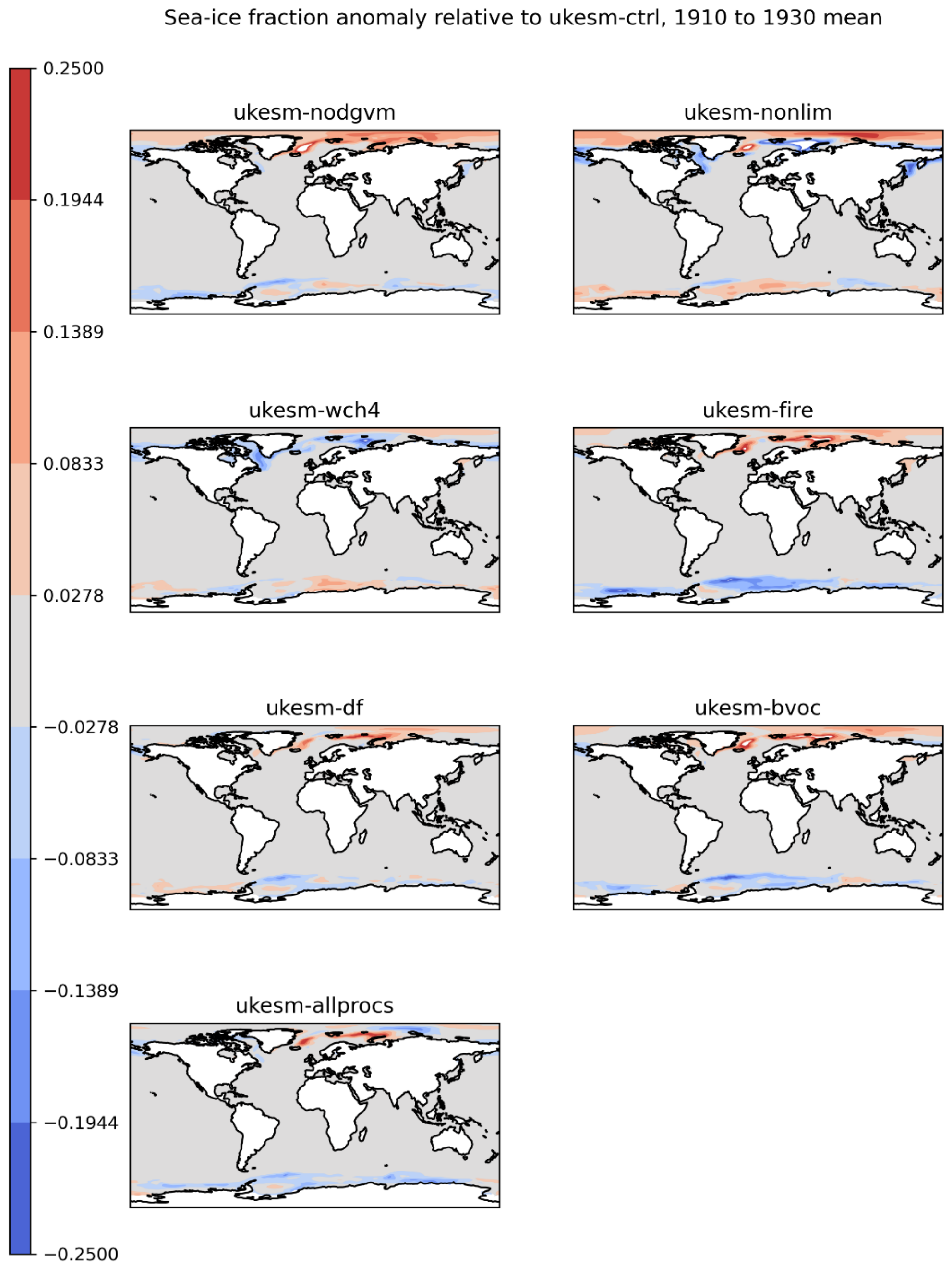

Supplementary Figure 12: Leaf Area Index (m^2 m^{-2}) anomaly of each configuration relative to *ukesm-ctrl* at 2xCO_2 in the fully coupled *1pctCO2* experiment, averaged over years 60 to 80 (1910 to 1930) and drift-corrected by subtracting the average of years 60 to 80 of the configuration-specific *piControl* simulation. The gridbox mean (GBM), the average of all plant functional types weighted by fraction, is shown.

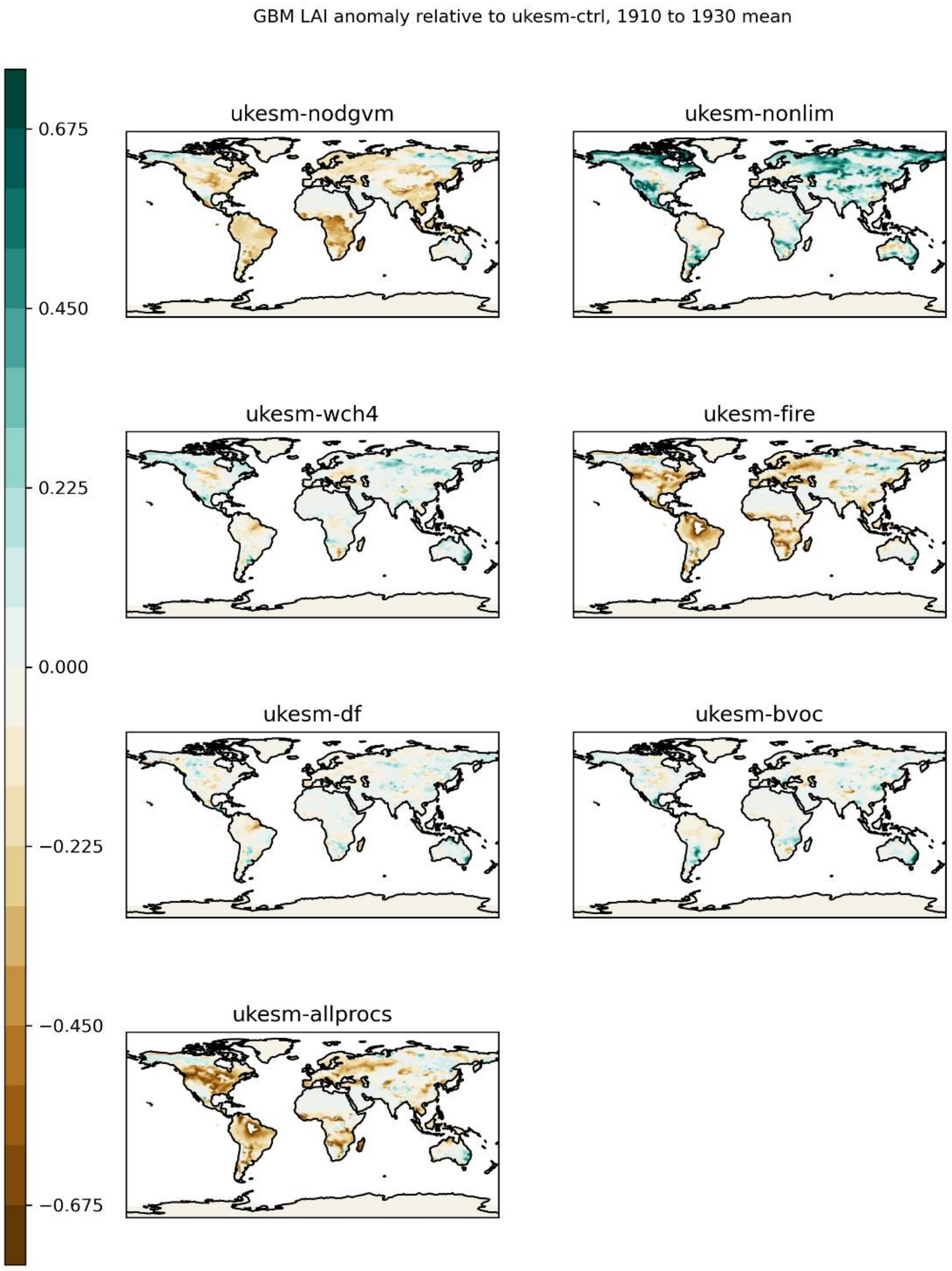

Supplementary Figure 13: Cumulative Ocean Heat Uptake anomaly (ZJ) relative to *ukesm-ctrl* during the 70 years in which CO_2 concentration doubles during the *1pctCO2* simulation (1850 to 1920), bias corrected for the configuration-specific *piControl* over the same period.

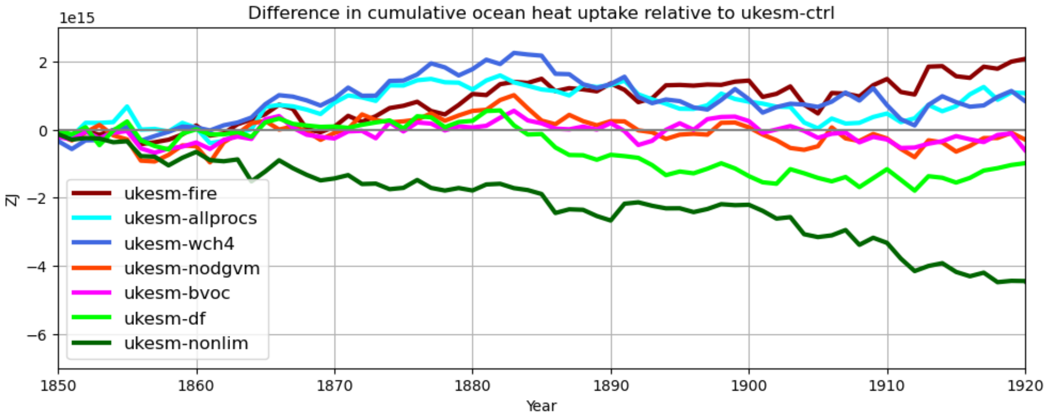

Supplementary Figure 14: Atlantic Meridional Overturning Circulation (AMOC) at 26.5N anomaly relative to *ukesm-ctrl*, smoothed with a 5 year running mean, drift-corrected by subtracting the average of the configuration-specific *piControl* simulation.

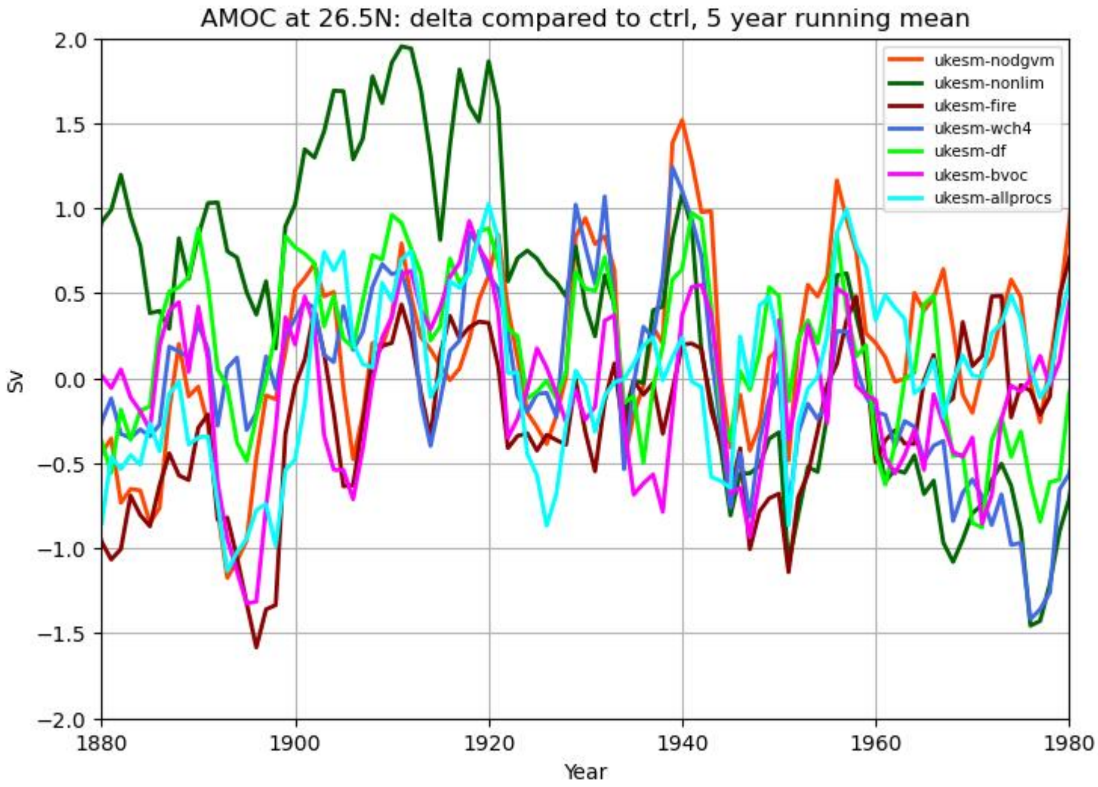

Supplement: Supplementary file 1 — Supplementary Information [file 41467_2026_72930_MOESM1_ESM.pdf]
