# Supplementary material for: Role of Earth system processes in the relationship between climate change and cumulative carbon emissions
Source: Nat Commun. 2026 May 19;17:6722. doi: 10.1038/s41467-026-72930-7 (PMC13385852; doi:10.1038/s41467-026-72930-7)
Supplement: Supplementary file 2 — Peer Review file [file 41467_2026_72930_MOESM2_ESM.pdf]

nature portfolio

Peer Review File

Role of Earth system processes in the relationship between climate change and cumulative carbon emissions.

Corresponding Author: Mr Spencer Liddicoat

This file contains all reviewer reports in order by version, followed by all author rebuttals in order by version.

Version 0:

Reviewer comments:

Reviewer #1

(Remarks to the Author)
This paper investigates the contribution of land carbon-cycle-related processes to the TCRE (via their change in temperature in UKESM and their computed impact on compatible cumulative emissions at a doubling of CO2 concentration in a 1pctCO2 experiment). It also frames these results in the context of the change in remaining carbon budget required to keep warming to under 1.5 degrees C vs 2 degrees C as well as in the context of the CMIP7 experiments that will likely include new process representation. Since the TCRE is an integral metric of the climate system, and the contribution of terrestrial carbon cycle processes to this metric will fundamentally improve our understanding of the Earth system, the motivation for this work is exciting and the results would be noteworthy. However, I have serious concerns about the methodology used to achieve these results and the resulting implications. My concerns are related to the presumed linearity of process-contribution of TCRE especially derived from one model and then applied to another in a concentration-driven experiment (without discussion of the weaknesses of that approach). This paper seeks to present relevant results in advance of CMIP7 experiments, which is a great idea to show the gravity of this work, but given the fact that larger differences between CMIP6 and CMIP7 (overshoot scenarios, emissions-driven runs), I'm concerned that the true fundamental importance of the contribution of biogeochemical processes to the sensitivity of the climate system is getting a bit lost in the attempt to connect it to global carbon budgets whose importance is quickly evolving/changing.

Major comments
• Methodology concerns – connecting non-UKESM ESMs. I have trouble understanding why this is paper is not solely concerned with the contribution of process-inclusion in UKESM and how it can have correspondence to other ESMs. The validation described in 137-143 might demonstrate an attribution you can make to a process within UKESM, but I do not think this demonstrates the efficacy of this technique for model to model comparison. Why are the other ESMs included in the results numbers when the differences between models include far more than whether these processes are included? This is particularly hard to interpret given that the ESMs are not labelled in the main article figures, and it necessary to relate Fig 3 to Table S3 and Table S2 to understand how the processes are implied to change the ESMs' TCREs. I guess I am not convinced that UKESM being an outlier in its TCRE is because it includes more land processes and not because it (also) represents those processes differently (structurally) given the low amount of carbon it stores in longer land carbon pools, deals with aerosols differently, has a weaker ocean sink, etc. and therefore that the scaling factors can be applied to them in the same way.
• Methodological/interpretation concerns of concentration-driven runs. The fact that these runs are concentration-driven has issues that are well-established, and so does the way ZEC is quantified. The authors claim to produce a take-away about individual process contribution to TCRE, but I'm not convinced that this methodology can be trusted in the context of the experimental design of the simulations. This required running a concentration-driven experiment omitting a process and then inferring the compatible cumulative FF CO2 emissions associated with that process based on the carbon sinks, but (1) that implies a difference in the rate of emissions for each experiment (2) each process seems use the same assumptions about land use emissions – but is this appropriate given the processes under consideration? It would be useful to at least address these methodological weaknesses directly especially when discussing implications for CMIP7 runs, given that they will be emissions-driven. This is a much larger change for the methodology of this paper than the inclusion of process representation between model versions, which is all that is currently discussed.
• Methodology concern – remaining carbon budget. I'm concerned that including eight different ways of calculating the RCB might create a false sense of coherence rather than just explaining one method and using it explicitly (and including the

values for all versions of UKESM and ESMs for that calculation), which may allow others to reproduce results more easily or use different methods. In fact the dominant change is going to be in changing the T_lim, and it's not clear to me that changing from zero to a near-zero number for ZEC (instead of changing the time horizon for when ZEC is measured (something that would change the result more dramatically) or referencing different approaches that have defined the RCB) is being recognized. Relatedly, perhaps a trivial question: Is it true that we can define the RCB change as the difference between T =1.5 and T = 2.0 as the delta_T instead of calculating the difference between RCB_1.5 and RCB_2.0? Perhaps a better way to build confidence would be to compare these results with studies of the processes themselves (existing bottom-up estimates or inferences from the literature).

- Framing with the RCB. Is the remaining carbon budget the most relevant implication to focus on? Since it's been shown that the T_lim = 1.5 degrees C RCB is almost zero and as overshoot scenarios become more the focal point of policy rather than 2 degrees C (for CMIP7) it might be worth either (1) including the implications for RCB only in a smaller way, and focusing on the fact that these processes change our a fundamental metric of the Earth system's response to forcing or (2) addressing the fact that moving up or down the TCRE may not be symmetric (relevant to lines 22-24, conclusions).
- Framing of TCRE change: It would be great to see the raw change in TCRE attributed to each process. As it is, I think the results are slightly vague, and it's hard to interpret exactly how each scaling factor can be compared to on another or even what the raw value of change in TCRE in the main part of the paper.

Minor comments

- Lines 13-15: since this is the definition of the TCRE, I think this sentence and lines 26-28 could be reworded to include CO2 in the definition of TCRE. Shouldn't it be the "Transient Climate Response to cumulative *CO2* emissions"? As it is, "CO2" is excluded from the long name. Also relevant for subsection header in line 84.
- Lines 175-179: This is a very difficult sentence to understand. Is the contribution of the combination of all of these processes what is being accounted for? It would be better to quantify things consistently (keep to only percent change, with or without uncertainty). There may also be a typo: "reduce" should be "reduces" in line 178.
- Line 179-181: This line is in future tense. When will this happen? In CMIP7? Perhaps introduce CMIP7 ahead of this sentence.
- Lines 190, 194, 359, 368 and elsewhere: Some of the terminology in this paper I believe is misused. For example, the use of the term "emulator" and "multiplicative scaling factors" are misleading when they are referring to a simple linear scaling, especially when we might expect these processes to be non-linear.
- Line 197 -199 seems to undercut the methodological claims in this study, attributing changes in the TCRE to "recent improvements and bias corrections" when changes in the TCRE between models is attributed to process inclusion alone.
- Lines 320-325 and elsewhere: I think it might be better to use "prognostic" than "emissions-driven" in the CH4 experiment description/identifier.
- Suggested title change to specify "land" carbon system processes influence on TCRE?

(Remarks on code availability)
There is no code or README provided, but I have reviewed the equations included.

Reviewer #2

(Remarks to the Author)
The authors present a new method for correcting the TCRE to account for missing processes in CMIP6 models, with the goal of bridging the gap to future CMIP7 estimates. The manuscript is well written, with clear explanations that are easy to follow. The figures are helpful and effectively support the text. The proposed approach -- revising the CMIP6 TCRE estimates using UKESM-derived scaling factors to capture the influence of missing processes -- is innovative, well-reasoned, and highly useful.

Yet, it is possible that the revised CMIP6 TCRE estimate derived from the approach in this study will not lie between the CMIP6 and CMIP7 values, but could instead be higher than the CMIP7 TCRE estimates. The method introduced here accounts for all currently missing processes (from the six analysed), whereas it is likely that not all CMIP7 models will have implemented these processes by the time CMIP7 TCRE results are produced. In addition, CMIP7 models may incorporate new processes that are not captured by the UKESM-based scaling factors. It would be worthwhile to briefly discuss the possibility that the revised CMIP6 TCRE estimates presented here may not actually bridge CMIP6 and CMIP7, and to explain why the approach nonetheless represents a valuable step forward.

Furthermore, it would be insightful to compare the UKESM-based scaling factors with analogous scaling factors derived from other ESMs -- or, alternatively, to show that applying the UKESM-derived scaling factors to other ESMs produces similarly good results as when applied to UKESM itself. However, this could be considered beyond the scope of the present study.

I therefore recommend the manuscript for publication in Nature Communications, with only minor comments to be addressed (see attached pdf).

Sabine Mathesius

(Remarks on code availability)

Version 1:

Reviewer comments:

Reviewer #2

(Remarks to the Author)
The authors provide a very interesting analysis on how different processes in an Earth system model (UKESM) influence the model's TCR and TCRE (Transient Climate Response to cumulative CO_2 Emissions). They demonstrate that the net effect of the six different processes is positive, i.e. modelled TCRE increases. The authors conclude from their results that the multimodel mean CMIP7 estimate of TCRE will be higher than the CMIP6 estimate, since many models will have incorporated some or all of the temperature-increasing processes. While this conclusion is based on UKESM results alone, it is valid to draw this conclusion, as the analysis focused on a general understanding of the individual process effects, which are qualitatively(!) likely not model dependent.

The detailed description of the methods would allow for reproducing the study, with UKESM or other Earth System Models. The modelling and process analysis seem robust and the results are relevant for a wide audience, I recommend the publication in Nature Communications.

A few minor suggestions (not hard requirements):

- line 19: I'd suggest adding in the abstract what motivated your study and why it is relevant beyond the UKESM community; i.e. mention briefly potential implications for future TCRE and carbon budget estimates.

- line 159: discuss how ocean carbon uptake might respond to the different scenarios if the simulations were not concentration-driven but emission-driven. Would you expect larger changes than in the concentration-driven runs and if so, how large?

- line 232: previous studies found a decrease in albedo when the global tree fraction increases (e.g. Matthews et al. 2022), due to the darker color of forests. How do you explain the opposite signal in UKESM? Does UKESM account for increased cloud cover over forests (which many other studies do not)?

- line 252: suppressed Southern Ocean deep water formation should also decrease Southern Ocean carbon uptake. It would be noteworthy that, in the real world or in an emission driven run, this would tend to increase atmospheric CO_2. In a concentration driven run, where ocean carbon uptake changes obviously don't affect the atmospheric CO_2 concentration, I would assume that a faster ocean-atmosphere equilibrium in the Southern Ocean (due to higher stratification) would at least affect the diagnosed emissions. The section on TCRE might not be the best place to address this - but a brief section on expected differences in an emission-driven runs, compared to the concentration-driven runs, would be valuable.

- line 303: Although beyond the scope of this study, it would be very interesting to see the quantitative effect of the different processes (or their sum) on the remaining carbon budget, in a follow-up study.

Open Access This Peer Review File is licensed under a Creative Commons Attribution 4.0 International License, which permits use, sharing, adaptation, distribution and reproduction in any medium or format, as long as you give appropriate credit to the original author(s) and the source, provide a link to the Creative Commons license, and indicate if changes were made.
In cases where reviewers are anonymous, credit should be given to 'Anonymous Referee' and the source.
The images or other third party material in this Peer Review File are included in the article's Creative Commons license, unless indicated otherwise in a credit line to the material. If material is not included in the article's Creative Commons license and your intended use is not permitted by statutory regulation or exceeds the permitted use, you will need to obtain permission directly from the copyright holder.
To view a copy of this license, visit https://creativecommons.org/licenses/by/4.0/

Reviewer #1 (Remarks to the Author):

This paper investigates the contribution of land carbon-cycle-related processes to the TCRE (via their change in temperature in UKESM and their computed impact on compatible cumulative emissions at a doubling of CO2 concentration in a 1pctCO2 experiment). It also frames these results in the context of the change in remaining carbon budget required to keep warming to under 1.5 degrees C vs 2 degrees C as well as in the context of the CMIP7 experiments that will likely include new process representation. Since the TCRE is an integral metric of the climate system, and the contribution of terrestrial carbon cycle processes to this metric will fundamentally improve our understanding of the Earth system, the motivation for this work is exciting and the results would be noteworthy. However, I have serious concerns about the methodology used to achieve these results and the resulting implications. My concerns are related to the presumed linearity of process-contribution of TCRE especially derived from one model and then applied to another in a concentration-driven experiment (without discussion of the weaknesses of that approach). This paper seeks to present relevant results in advance of CMIP7 experiments, which is a great idea to show the gravity of this work, but given the fact that larger differences between CMIP6 and CMIP7 (overshoot scenarios, emissions-driven runs), I'm concerned that the true fundamental importance of the contribution of biogeochemical processes to the sensitivity of the climate system is getting a bit lost in the attempt to connect it to global carbon budgets whose importance is quickly evolving/changing.

SKL: Thank you for taking the time to review the paper so thoroughly. We accept your comments and have addressed your main methodological concerns primarily by reframing the paper to focus on the UKESM TCRE results, rather than applying them numerically to the other CMIP6 ESMs. The thrust of the paper is now a deeper dive into understanding and explaining the differences in TCRE across the UKESM process-ensemble. Therefore, there is much less of a focus on the remaining carbon budget, though we still discuss the relationship between TCRE and carbon budgets to future waring targets in the context of our results.

Major comments

• Methodology concerns – connecting non-UKESM ESMs. I have trouble understanding why this is paper is not solely concerned with the contribution of process-inclusion in UKESM and how it can have correspondence to other ESMs. The validation described in 137-143 might demonstrate an attribution you can make to a process within UKESM, but I do not think this demonstrates the efficacy of this technique for model to model comparison. Why are the other ESMs included in the results numbers when the differences between models include far more than whether these processes are included? This is particularly hard to interpret given that the ESMs are not labelled in the main article figures, and it necessary to relate Fig 3 to Table S3 and Table S2 to understand how the processes are implied to change the ESMs' TCREs. I guess I am not convinced that UKESM being an outlier in its TCRE is because it includes more land processes and not because it (also) represents those processes differently (structurally) given the low amount of carbon it stores in longer land carbon pools, deals with aerosols differently, has a weaker ocean sink, etc. and therefore that the scaling factors can be applied to them in the same way.

SKL: The paper is now largely focused on the TCRE results of the UKESM process ensemble. We expand on the original TCR vs Airborne Fraction plot by discussing in much greater depth why

the AF and TCR deviate from those of ukesm-ctrl, and quantify how each of these contributes to the TCRE differences. We no longer attempt to apply our results numerically to the CMIP6 ESMs but discuss the potential implications for TCRE at CMIP7 as some of the processes are expected to become more prevalent at CMIP7 – we have added two recent references to provide further evidence for this. We also explicitly relate our results back to those of Arora et al (2020), discussing their N-limited versus non-N-limited TCREs in the context of our experiments.

• Methodological/interpretation concerns of concentration-driven runs. The fact that these runs are concentration-driven has issues that are well-established, and so does the way ZEC is quantified. The authors claim to produce a take-away about individual process contribution to TCRE, but I'm not convinced that this methodology can be trusted in the context of the experimental design of the simulations. This required running a concentration-driven experiment omitting a process and then inferring the compatible cumulative FF CO2 emissions associated with that process based on the carbon sinks, but (1) that implies a difference in the rate of emissions for each experiment (2) each process seems use the same assumptions about land use emissions – but is this appropriate given the processes under consideration? It would be useful to at least address these methodological weaknesses directly especially when discussing implications for CMIP7 runs, given that they will be emissions-driven. This is a much larger change for the methodology of this paper than the inclusion of process representation between model versions, which is all that is currently discussed.

SKL: The 1pctCO2 experiments have been the standard approach to calculating TCRE from within ESMs since its inception - the diagnosed compatible CO2 emissions rate is inherently unique to each ESM, or in our case, UKESM configuration, as it is a function of the land and ocean carbon sinks: these, particularly the land carbon sink, are how the process changes in our study influence the TCRE, to a large extent. The key point is that all the ESMs / UKESM configurations use the same transient CO2 profile to drive the sinks.

The land use forcing is the same in each of our UKESM configurations – the 1850 land use distribution is applied throughout the 1pctCO2 and piControl simulations. In UKESM this means that the crop and pasture plant functional types (PFTs) are not allowed to exceed the 1850 land use distribution and encroach onto the part of the grid box reserved for natural vegetation. Since the crop and pasture PFTs don't necessarily occupy the entirety of the region in which they are permitted to grow at the start of the simulations, there can be some change to the crop and pasture PFT fractions as the CO2 concentration and climate change. This will impact the crop harvest flux, which is a function of the crop PFT fraction and the crop productivity, and varies across configurations. Hence the land use forcing applied to each configuration is the same in all cases, but the impact of that imposed forcing – the land use emissions (primarily the harvest flux), which act to weaken the land carbon sink - is configuration-specific and influences the compatible CO2 emissions and therefore the TCRE.

The 1pctCO2 runs we performed for this study will be replicated at CMIP7, exactly; the future scenarios will be CO2 emissions-driven, but the concentration-driven 1pctCO2 experiments remain part of the DECK at CMIP7 exactly as they were at CMIP6, partly for the quantification of climate - carbon cycle feedback parameters but also for TCRE.

• Methodology concern – remaining carbon budget. I'm concerned that including eight different ways of calculating the RCB might create a false sense of coherence rather than just explaining one method and using it explicitly (and including the values for all versions of UKESM and ESMs for that calculation), which may allow others to reproduce results more easily or use different

methods. In fact the dominant change is going to be in changing the T_lim, and it's not clear to me that changing from zero to a near-zero number for ZEC (instead of changing the time horizon for when ZEC is measured (something that would change the result more dramatically) or referencing different approaches that have defined the RCB) is being recognized. Relatedly, perhaps a trivial question: Is it true that we can define the RCB change as the difference between T =1.5 and T = 2.0 as the delta_T instead of calculating the difference between RCB_1.5 and RCB_2.0? Perhaps a better way to build confidence would be to compare these results with studies of the processes themselves (existing bottom-up estimates or inferences from the literature).

SKL: These are useful comments that we will think about if we explore this side of the work further in future, thank you. In the present manuscript, we have removed the remaining carbon budget analysis referred to here, so this methodological concern is no longer an issue.

• Framing with the RCB. Is the remaining carbon budget the most relevant implication to focus on? Since it's been shown that the T_lim = 1.5 degrees C RCB is almost zero and as overshoot scenarios become more the focal point of policy rather than 2 degrees C (for CMIP7) it might be worth either (1) including the implications for RCB only in a smaller way, and focusing on the fact that these processes change our a fundamental metric of the Earth system's response to forcing or (2) addressing the fact that moving up or down the TCRE may not be symmetric (relevant to lines 22-24, conclusions).

SKL: We agree, and have removed much of what this comment referred to. We still refer to the implications that changes in TCRE might have on the RCB, i.e. if the CMIP7 ESMs' TCRE does increase relative to the CMIP6 ESMs as more processes are included, that implies the remaining carbon budgets to future warming targets will reduce – but to a much lesser extent than we did in the original manuscript,

• Framing of TCRE change: It would be great to see the raw change in TCRE attributed to each process. As it is, I think the results are slightly vague, and it's hard to interpret exactly how each scaling factor can be compared to on another or even what the raw value of change in TCRE in the main part of the paper.

SKL: Thank you, we've provided a much more thorough explanation as to the underlying causes behind the TCRE changes: quantifying the relative importance of AF vs TCR changes, and why AF and TCR change the way they do. We have included additional plots on the land and ocean carbon cycle response and, in Supplementary Information, maps of anomalies in physical fields between the experimental configurations and ukesm-ctrl, which help to explain the TCR differences. We agree that the paper is better served by going into much greater detail here, which potentially provides more clarity in understanding how applicable the UKESM response is to other ESMs. For example, we discuss how some configurations change the atmospheric composition and how this affects TCR, whereas other configurations don't explicitly change the atmospheric composition, so change the TCR primarily via biophysical feedbacks to climate. We use the regression method of Andrews and Ringer (2014) (now both co-authors) to diagnose the climate feedback parameter. Using an expression for TCR derived from the energy budget equation, we discuss the influence on TCR of the climate feedback parameter, effective radiative forcing and ocean heat uptake to explain the highest and lowest TCRs of our UKESM ensemble.

Minor comments

• Lines 13-15: since this is the definition of the TCRE, I think this sentence and lines 26-28 could be reworded to include CO_2 in the definition of TCRE. Shouldn't it be the "Transient Climate Response to cumulative *CO2* emissions"? As it is, "CO2" is excluded from the long name. Also relevant for subsection header in line 84.

SKL: Thank you, now changed.

• Lines 175-179: This is a very difficult sentence to understand. Is the contribution of the combination of all of these processes what is being accounted for? It would be better to quantify things consistently (keep to only percent change, with or without uncertainty). There may also be a typo: "reduce" should be "reduces" in line 178.

SKL: This sentence is no longer present.

• Line 179-181: This line is in future tense. When will this happen? In CMIP7? Perhaps introduce CMIP7 ahead of this sentence.

SKL: This section has been rewritten, so those lines no longer exist.

• Lines 190, 194, 359, 368 and elsewhere: Some of the terminology in this paper I believe is misused. For example, the use of the term "emulator" and "multiplicative scaling factors" are misleading when they are referring to a simple linear scaling, especially when we might expect these processes to be non-linear.

SKL: Thank you for pointing out that lack of clarity. Now that we have removed the section regarding rescaling the CMIP6 ESMs' TCREs, those terms now no longer exist in the manuscript.

• Line 197 -199 seems to undercut the methodological claims in this study, attributing changes in the TCRE to "recent improvements and bias corrections" when changes in the TCRE between models is attributed to process inclusion alone.

SKL: This refers to the differences between the earlier version of UKESM (UKESM1-0-LL) and the version used in this study (UKESM1.1). We're aiming to highlight the importance of certain processes in isolation on TCRE, rather than claiming that process differences are solely responsible for the differences in model behaviour.

• Lines 320-325 and elsewhere: I think it might be better to use "prognostic" than "emissions-driven" in the CH_4 experiment description/identifier.

SKL: That's a fair point. We've added 'prognostic' in one instance but left the other as "having emissions-driven methane capability".

• Suggested title change to specify "land" carbon system processes influence on TCRE?

SKL: We've changed the title to reflect the refocus on the mechanistic differences underlying the TCRE changes rather than carbon budget. We've kept "Earth system" rather than changing to "land carbon" as the processes are all Earth System process, and some are explicit land carbon processes, but others could be more accurately said to be related to land carbon processes.

Reviewer #1 (Remarks on code availability):

There is no code or README provided, but I have reviewed the equations included.

SKL: Apologies, I attempted to upload the code which performs the scaling of the CMIP6 ESMs' TCREs and the associated carbon budget calculations when uploading the manuscript originally, but that obviously didn't work. Thank you for reviewing the equations.

Reviewer #2 (Remarks to the Author):

SKL: Thank you for taking the time to review the paper, Dr. Mathesius, and for your positive comments regarding the manuscript.

The authors present a new method for correcting the TCRE to account for missing processes in CMIP6 models, with the goal of bridging the gap to future CMIP7 estimates. The manuscript is well written, with clear explanations that are easy to follow. The figures are helpful and effectively support the text. The proposed approach -- revising the CMIP6 TCRE estimates using UKESM-derived scaling factors to capture the influence of missing processes -- is innovative, well-reasoned, and highly useful.

SKL: Following other reviewers' comments, you will see that we have reframed the paper to focus on the process-induced differences in TCRE in UKESM, rather than applying numerically UKESM's results to the other CMIP6 ESMs. We go deeper into explaining why the processes impact the TCRE in UKESM in the way that they do: we present numerically the contribution the process-induced TCR and Airborne Fraction differences make to the change in TCRE relative to the control configuration, and discuss underlying mechanisms. We show and discuss the spread in land and ocean carbon uptake across the UKESM ensemble which cause their Airborne Fraction differences. In the case of nitrogen limitation of land carbon uptake, we relate our results back to those of Arora et al (2020), discussing their N-limited versus non-N-limited TCREs in the context of our experiments.

For the TCR, we discuss the changes in atmospheric composition which contribute to the TCR differences in several configurations. We adopt the regression method of Andrews and Ringer (2014), both now co-authors, to calculate the climate feedback parameter which we use in an expression for TCR to help to explain the spread in TCR and the apparent underlying physical mechanisms.

Our conclusions remain the same: that the increase in the prevalence of processes like interactive wetland methane emissions and fire from CMIP6 to CMIP7 is likely to lead to an increase in TCRE at CMIP7, in the absence of any counteracting physical changes which exert a cooling influence. We relate this to the implications for future carbon budgets – if the TCRE does increase at CMIP7, the inverse relationship between the TCRE and the remaining carbon budgets implies the latter will reduce – without taking the step to quantify numerically how the TCRE and carbon budgets might change based on our UKESM results.

Since the TCRE is the primary determinant of the magnitude of future remaining carbon budgets, we think refocusing our manuscript towards a deeper dive into the process-induced changes to TCRE in the present draft is justified.

Yet, it is possible that the revised CMIP6 TCRE estimate derived from the approach in this study will not lie between the CMIP6 and CMIP7 values, but could instead be higher than the CMIP7 TCRE estimates. The method introduced here accounts for all currently missing processes (from the six analysed), whereas it is likely that not all CMIP7 models will have implemented these processes by the time CMIP7 TCRE results are produced. In addition, CMIP7 models may incorporate new processes that are not captured by the UKESM-based scaling factors. It would be worthwhile to briefly discuss the possibility that the revised CMIP6 TCRE estimates presented here may not actually bridge CMIP6 and CMIP7, and to explain why the approach nonetheless represents a valuable step forward.

SKL: This is a good point, thank you. Yes, it is unlikely that all ESMs will adopt all six processes we discuss, and no doubt some will include other processes, perhaps carbon loss from permafrost thaw or from drying peatland, which will act to increase TCRE relative to their CMIP6 version. Since we have now removed the scaling factor approach, we don't quantify numerically how the TCRE might change if the ESMs were to include all six processes, but we still talk about the 'direction of travel' of TCRE from CMIP6 to CMIP7 as processes such as interactive wetland methane emissions and fire become more common.

Furthermore, it would be insightful to compare the UKESM-based scaling factors with analogous scaling factors derived from other ESMs -- or, alternatively, to show that applying the UKESM-derived scaling factors to other ESMs produces similarly good results as when applied to UKESM itself. However, this could be considered beyond the scope of the present study.

SKL: Thank you, this would have been very worthwhile if we had kept the scaling factor results.

I therefore recommend the manuscript for publication in Nature Communications, with only minor comments to be addressed (see attached pdf).

SKL: Many thanks again for taking the time to go through the manuscript so thoroughly, and for your recommendations. Thank you for you feedback on our scaling factor approach, we will take your comments and suggestions into account if we revisit this work in future.

Sabine Mathesius

REVIEWERS' COMMENTS

Reviewer #2 (Remarks to the Author):

The authors provide a very interesting analysis on how different processes in an Earth system model (UKESM) influence the model's TCR and TCRE (Transient Climate Response to cumulative CO2 Emissions). They demonstrate that the net effect of the six different processes is positive, i.e. modelled TCRE increases. The authors conclude from their results that the multimodel mean CMIP7 estimate of TCRE will be higher than the CMIP6 estimate, since many models will have incorporated some or all of the temperature-increasing processes. While this conclusion is based on UKESM results alone, it is valid to draw this conclusion, as the analysis focused on a general understanding of the individual process effects, which are qualitatively(!) likely not model dependent.

The detailed description of the methods would allow for reproducing the study, with UKESM or other Earth System Models. The modelling and process analysis seem robust and the results are relevant for a wide audience, I recommend the publication in Nature Communications.

Many thanks for your continued effort in reviewing the resubmitted paper and for your positive comments.

A few minor suggestions (not hard requirements):

- line 19: I'd suggest adding in the abstract what motivated your study and why it is relevant beyond the UKESM community; i.e. mention briefly potential implications for future TCRE and carbon budget estimates.

We have added a couple of sentences to the abstract to reflect this. It now reads, in full:

Estimates of carbon emissions budgets to limit global warming to 1.5°C or 2°C rely on the near-linear relationship between global temperature change and total CO_2 emitted, known as the Transient Climate Response to cumulative CO_2 Emissions (TCRE). The TCRE is determined from Earth System Models (ESMs) and is therefore sensitive to the physical and biogeochemical processes represented within them. Here we use an ESM (UKESM) to explore the sensitivity of TCRE to six Earth system processes in isolation. Four processes increase TCRE: fire-vegetation interactions by 14.6%; nitrogen limitation of vegetation by 9.7%; diffuse radiation effects on vegetation by 8.5%; and interactive emissions of methane from wetlands by 5.1%. Conversely, two processes marginally reduce TCRE: allowing the vegetation distribution to adapt to changing climate and CO_2 lowers TCRE by 1.5%, and climate impacts from the emission of biogenic volatile organic compounds reduce it by 1.4%. We demonstrate the extent to which each process changes TCRE via its influence on the climate and on the global carbon cycle, and discuss underlying mechanisms. Our results highlight the substantial process-dependence of model-derived estimates of TCRE, with implications for remaining carbon budgets to future warming targets calculated from them.

- line 159: discuss how ocean carbon uptake might respond to the different scenarios if the simulations were not concentration-driven but emission-driven. Would you expect larger changes than in the concentration-driven runs and if so, how large?

We have added a paragraph to the Discussion on the emissions-driven vs concentration-driven point.

"The process-induced changes in TCRE presented here are diagnosed from CO_2 concentration driven experiments. In CO_2 emissions-driven mode, the large changes in airborne fraction observed in some configurations would equate to potentially significant changes in CO_2-induced radiative forcing, widening further the spread in TCRE. For example, including fire-vegetation interactions increased markedly the airborne fraction: in emissions-driven mode, this would translate into a higher CO_2 concentration relative to the control configuration for a given emissions scenario, driving up TCRE. Conversely, disabling nitrogen limitation, promoting land carbon uptake, would reduce atmospheric CO_2 relative to *ukesm-ctrl*, delaying the point at which CO_2 concentration doubles, thereby lowering TCRE. Hence, the process-induced spread in TCRE observed in our concentration-driven experiments may be somewhat conservative."

- line 232: previous studies found a decrease in albedo when the global tree fraction increases (e.g. Matthews et al. 2022), due to the darker color of forests. How do you explain the opposite signal in UKESM? Does UKESM account for increased cloud cover over forests (which many other studies do not)?

I think our results are consistent with the change seen in Matthews et al (2022). The blue colour of the northern hemisphere land in the albedo anomaly figure of *ukesm-nodgvm* (Supplementary Figure 4) indicates higher albedo (the colour scheme was chosen such that higher albedo than *ukesm-ctrl*, having a cooling effect, is blue, with lower albedo regions being coloured red). This is consistent with *ukesm-ctrl* having a higher tree fraction, being darker and having lower albedo than *ukesm-nodgvm*, hence the difference plot showing large blue regions over land.

- line 252: suppressed Southern Ocean deep water formation should also decrease Southern Ocean carbon uptake. It would be noteworthy that, in the real world or in an emission driven run, this would tend to increase atmospheric CO2. In a concentration driven run, where ocean carbon uptake changes obviously don't affect the atmospheric CO2 concentration, I would assume that a faster ocean-atmosphere equilibrium in the Southern Ocean (due to higher stratification) would at least affect the diagnosed emissions. The section on TCRE might not be the best place to address this - but a brief section on expected differences in an emission-driven runs, compared to the concentration-driven runs, would be valuable.

The new paragraph in the Discussion section addresses the emission-driven vs concentration-driven point. Your comment about differences in Southern Ocean carbon uptake between the two is likely correct, but we don't specifically mention the Southern Ocean in the new paragraph, as it's difficult to comment on the magnitude of potential differences there without having done parallel experiments.

- line 303: Although beyond the scope of this study, it would be very interesting to see the quantitative effect of the different processes (or their sum) on the remaining carbon budget, in a follow-up study.

Thank you for your interest. The (now removed) section of the first version of this study which applied the influence of the processes absent from the CMIP6 ESMs to their TCRE, after which we calculated how the processes influence the carbon budget from 1.5°C to 2.0°C, has been improved and extended to be a separate study in its own right. As of 1st April 2026, this is under review at Nature Communications Earth and Environment.
